# Effects of different electrical stimulation on depression: a systematic review and network meta-analysis

**DOI:** 10.3389/fpsyt.2026.1962608

**Published:** 2026-10-27

**Authors:** Jiajin Chai, Song Li, Junhao Huang, Shaoyan Yao, Lei Zhou, Xichen Chen, Fei Liu, Changliang Ji, Hanyu Chen, Shu Tan, Rui Li, Runjuan Ma, Shikun Li, Shouping Zhao

**Affiliations:** 1 The Affiliated Mental Health Center of Kunming Medical University, Kunming, China; 2 The Second Clinical Medical College, Heilongjiang University of Chinese Medicine, Harbin, China; 3 Yunnan University of Chinese Medicine, Kunming, China; 4 The First Affiliated Hospital of Kunming Medical University, Kunming, China; 5 The Second School of Clinical Medicine and The Second Affiliated Hospital of Kunming Medical University, Kunming, China

**Keywords:** depression, electrical stimulation, network meta-analysis, randomised controlled trial, systematic review

## Abstract

**Background:**

Depression is a prevalent psychiatric disorder characterised by persistent low mood and anhedonia, substantially impairing quality of life. Conventional treatments show variable efficacy and tolerability, with high post-discontinuation relapse rates. Electrical stimulation has emerged as an alternative intervention, but comparative efficacy and safety of different modalities remain uncertain. This systematic review and network meta-analysis aimed to synthesise evidence and quantitatively compare electrical stimulation approaches for depression.

**Methods:**

A systematic search of 13 databases was conducted for randomised controlled trials (RCTs) published from inception to 16 July 2026. Methodological quality and risk of bias were assessed using the Cochrane RoB 2 tool, and certainty of evidence was evaluated with the GRADE framework. Frequentist network meta-analysis was performed using STATA 18.0. Publication bias was assessed via funnel plot inspection, and sensitivity analyses examined the robustness of findings.

**Results:**

A total of 33 RCTs involving 2,664 participants and nine electrical stimulation modalities were included. Pairwise meta-analysis showed that electrical stimulation was associated with a significantly improved clinical response rate (OR = 2.78, 95% CI 1.50 to 5.16) and a reduction in depressive symptom severity (SMD = −0.90, 95% CI −1.27 to −0.54) among patients with depression, along with a favourable safety profile (OR = 0.88, 95% CI 0.52 to 1.49). However, therapeutic effects varied across modalities. Specifically, Transcutaneous auricular vagus nerve stimulation (taVNS) ranked highest for improving the clinical response rate (SUCRA = 99.9%; OR = 4.12, 95% CI 3.08 to 5.16), whereas transcranial direct current stimulation (tDCS) ranked highest for reducing the Hamilton Depression Rating Scale (HAMD) score (SUCRA = 71.3%; SMD = −1.70, 95% CI −2.80 to −0.60). Sensitivity analyses confirmed the robustness of these results.

**Conclusions:**

Electrical stimulation is associated with improved clinical response rates and reduced depressive symptom severity in patients with depression, and is generally well tolerated. Among the modalities examined, taVNS demonstrated a relative advantage in improving clinical response, while tDCS showed a relative advantage in alleviating depressive symptom severity. However, given the low certainty of the evidence underpinning these findings, they require confirmation in larger, higher-quality randomised controlled trials.

**Systematic Review Registration:**

[url], identifier CRD420251103264.

## 1 Introduction

Depression remains among the most prevalent and disabling mental disorders worldwide. According to the 2019 Global Burden of Disease (GBD) study, approximately 280 million people are affected, and depression ranks second across all age groups in terms of years lived with disability (1). The COVID-19 pandemic has further intensified the global burden of depressive disorders, with the number of cases estimated to have risen by 27.6% by 2022 (2). Core clinical features include persistent low mood, anhedonia, cognitive impairment, and neurovegetative symptoms, which profoundly compromise psychosocial functioning, occupational productivity, and health-related quality of life (3). Although first-line pharmacological treatments—including selective serotonin reuptake inhibitors (SSRIs), serotonin–norepinephrine reuptake inhibitors (SNRIs), and tricylclic antidepressants—and evidence-based psychotherapies are widely implemented, approximately 30% of patients fail to achieve adequate response, a condition associated with disproportionately high rates of disability, healthcare service utilisation, and suicide risk (4, 5). Moreover, pharmacotherapy is frequently complicated by poor tolerability, with adverse effects such as weight gain, sexual dysfunction, emotional blunting, and gastrointestinal discomfort undermining treatment adherence and precipitating early discontinuation (6, 7). These persistent challenges have spurred the exploration of alternative neuromodulation-based interventions with distinct mechanisms of action.

Bioelectronic medicine is an emerging field that uses electrical signals to interface with and modulate the nervous system, and it has emerged as a promising strategy for neuropsychiatric disorders. Electrical stimulation techniques within this field can directly target dysfunctional neural circuits implicated in the pathophysiology of depression, including the dorsolateral prefrontal cortex (DLPFC), the subgenual anterior cingulate cortex (sgACC), and the limbic–cortical–striatal–pallidal–thalamic network (8, 9). These modalities span a broad spectrum with respect to invasiveness, mode of action at the target site, and clinical applicability. Electroconvulsive therapy (ECT) remains the most effective acute intervention for severe and treatment-resistant depression; contemporary practice employs various electrode configurations—such as bilateral temporal (BT), bilateral frontal (BF), high-dose unilateral (HUL), and low-dose unilateral (LUL)—to achieve a differentiated balance between antidepressant efficacy and cognitive tolerability (10, 11). Transcranial direct current stimulation (tDCS) delivers low-intensity (1–2 mA) direct current via scalp electrodes to induce polarity-dependent, subthreshold modulation of cortical excitability; its portability, low cost, and favourable safety profile enable application in both clinical settings and remotely supervised home environments (12–15). Transcranial alternating current stimulation (tACS) applies oscillatory currents at specific frequencies (typically within the α or γ bands) to entrain abnormal endogenous neural oscillations hypothesised to underlie depression (16, 17). Cranial electrotherapy stimulation (CES) uses pulsed, low-intensity microcurrents and has been associated with modulation of the hypothalamic–pituitary–adrenal (HPA) axis and brain-derived neurotrophic factor (BDNF) levels (18). Vagus nerve stimulation (VNS) encompasses two forms: an implantable device targeting the left cervical vagus nerve and the non-invasive transcutaneous auricular vagus nerve stimulation (taVNS). Both modalities deliver chronic, intermittent stimulation of vagal afferent pathways and have obtained regulatory approval for the treatment of depression in multiple jurisdictions (19). Transcutaneous acupoint electrical stimulation (TEAS), which integrates peripheral nerve modulation with traditional acupoint targeting, has recently emerged as an exploratory intervention for depression (20). Despite this diversity of available modalities, direct head-to-head comparative evidence to guide treatment selection remains critically lacking.

Although prior conventional meta-analyses and network meta-analyses (NMAs) have evaluated the clinical efficacy of electrical stimulation for depression (21–24), the existing evidence has focused predominantly on non-invasive brain stimulation techniques such as repetitive transcranial magnetic stimulation (rTMS) and tDCS. A unified comparative framework encompassing electrical stimulation therapies as a distinct therapeutic category is absent. Consequently, the current evidence base is insufficient to inform cross-modality clinical decision-making among electrical stimulation approaches. To address this gap, the present study pursued the following objectives (1): to determine the effects of electrical stimulation on clinical response rates and depressive symptom severity in patients with depression, and (2) to characterise the safety profile of electrical stimulation across modalities. By identifying the comparative effectiveness and tolerability of distinct electrical stimulation strategies, these findings are intended to support more informed clinical decision-making.

## 2 Materials and methods

The protocol for this NMA has been registered with PROSPERO (CRD420251103264). Following group discussion, several aspects of the methodology were refined. The primary outcomes are the clinical response rate and the HAMD score; we further restricted the control condition to sham electrical stimulation and updated the search cut-off date to 16 July 2026. This study was conducted in full accordance with the PRISMA 2020 statement (25) and its network meta-analysis extension (PRISMA-NMA) (26); the completed PRISMA-NMA checklist is provided in **Supplementary Digital Content 1**.

### 2.1 Search strategy

We searched 13 databases—PubMed, Embase, the Cochrane Library, Web of Science, Scopus, ClinicalTrials.gov, the WHO International Clinical Trials Registry Platform (WHO-ICTRP), OpenGrey, ProQuest, the China National Knowledge Infrastructure (CNKI), the VIP Database, the Wanfang Database, and the China Biomedical Database—from the inception of each database to 16 July 2026. Reference lists of previously included studies and published systematic reviews on this topic were manually screened to identify, supplement, and further assess relevant literature. Search terms combined subject headings (MeSH terms where applicable) and free-text terms. The detailed search strategy is presented in **Supplementary Digital Content 2**.

### 2.2 Inclusion and exclusion criteria

#### 2.2.1 Inclusion criteria

Studies meeting all of the following criteria were eligible for inclusion:

1. Participants: Participants were required to have a diagnosis of depression established by a qualified psychiatrist in accordance with the diagnostic criteria specified in the International Classification of Diseases, 11th Revision (ICD-11) or the Diagnostic and Statistical Manual of Mental Disorders, Fifth Edition, Text Revision (DSM-5-TR).
2. Interventions: Electrical stimulation modalities, including but not limited to ECT, CES, tACS, tDCS, VNS, taVNS and TEAS.
3. Comparators: The control group received sham electrical stimulation or an alternative electrical stimulation therapy.
4. Outcomes: The study explicitly reported at least one of the following: clinical response rate, HAMD score, or safety outcomes.
5. Study design: Randomised controlled trial (RCT).

#### 2.2.2 Exclusion criteria

Studies meeting any of the following criteria were excluded: (1) conference abstracts, animal studies, duplicate publications, systematic reviews, or meta-analyses; (2) studies for which data required for effect size calculation were unavailable, missing, or whose full text could not be retrieved despite contacting the corresponding author; (3) non-randomised controlled trials; and (4) studies in which participants in either the intervention or control arm received concomitant pharmacological or non-pharmacological treatments beyond the electrical stimulation protocol.

### 2.3 Outcome measures

Three commonly reported outcome domains—clinical response rate, depressive symptom severity as measured by the HAMD, and safety—were selected to evaluate the efficacy and tolerability of electrical stimulation therapy in patients with depression.

The clinical response rate was assessed using the Hamilton Depression Rating Scale (HDRS) and defined as the proportion of participants who achieved a positive treatment response at the study endpoint (Clinical Response Rate = Number of Responders/Total Number of Participants). The HDRS is a widely validated instrument for quantifying the severity of depressive symptoms, particularly in trials evaluating pharmacological and psychotherapeutic interventions. This study employed the 17-item version (HAMD-17), which yields a total score ranging from 0 to 50, with higher scores indicating greater symptom severity.

Safety outcomes primarily comprised adverse events (AEs), including the number of participants experiencing clinical symptoms such as palpitations, nausea, and vomiting.

### 2.4 Data collection

Two independent reviewers screened the literature using EndNote (version 20). Duplicate records were first identified through automated detection by the software and subsequently verified and removed manually. An initial screening of titles and abstracts was then conducted, followed by full-text assessment of records deemed potentially eligible after the initial screen. Upon completion of this process, the two reviewers cross-checked the included studies to confirm whether they met the predefined eligibility criteria. Relevant data were entered into Microsoft Excel (version 2019) for management. A structured data extraction form was developed based on the characteristics of the included studies. The principal information extracted comprised: (1) study characteristics—authors, year of publication, sample size, age, illness duration, and sex distribution; (2) study design, study duration, and outcome measures; and (3) specific interventions administered in the experimental and control arms. Where data reported in the original study were insufficient or key information was unclear, corresponding authors were contacted by email to obtain supplementary data. Throughout the screening and data extraction process, any discrepancies between the two reviewers were resolved through discussion with a third reviewer until consensus was reached. For studies that did not directly report the standard deviation (SD), it was derived from the standard error (SE), confidence interval (CI), t-value, or P-value as available. When none of the above statistics were reported, and for studies presenting data exclusively in graphical form, GetData Graph Digitizer (version 2.20) was used to extract the required values from the figures.

### 2.5 Risk of bias and quality of evidence grading

Two independent reviewers assessed the risk of bias of each included study using Version 2 of the Cochrane Risk of Bias tool (RoB 2) (27). RoB 2 comprises five domains: bias arising from the randomisation process, bias due to deviations from the intended intervention, bias due to missing outcome data, bias in the measurement of the outcome, and bias in the selection of the reported result. The assessment of each domain informs an overall risk-of-bias judgement. Following synthesis across all domains, the overall risk of bias is classified as “low risk of bias,” “some concerns,” or “high risk of bias.

In addition, two independent reviewers applied the Grading of Recommendations, Assessment, Development, and Evaluation (GRADE) framework to rate the certainty of evidence for each outcome across six dimensions: study design, risk of bias, inconsistency, indirectness, imprecision, and other considerations (28). To minimise bias, the review team received detailed guidance and standardised training before formally commencing the study. Any disagreements that arose during the assessment process were resolved through discussion within the expert panel.

### 2.6 Assessment of transitivity

The validity of indirect comparisons rests on the transitivity assumption, which requires that the distribution of effect modifiers be comparable across the trials informing the different comparisons in the network. To evaluate this assumption, we compared a predefined set of clinical and methodological characteristics considered likely to modify treatment effects, including baseline depression severity, the proportion of participants with treatment-resistant depression (TRD), mean age, duration of illness, treatment duration, and outcome evaluation period.

### 2.7 Statistical analysis

All statistical analyses were performed using Stata 18.0. For continuous outcomes, treatment effects were expressed as standardised mean differences (SMDs) with 95% confidence intervals (CIs); for dichotomous outcomes, odds ratios (ORs) with 95% CIs were used. Between-study heterogeneity was assessed using the chi-squared test and the I² statistic. In pairwise meta-analyses, a fixed-effects model was applied when heterogeneity was not significant (P > 0.1 or I² < 50%), whereas a random-effects model was adopted when significant heterogeneity was detected (P ≤ 0.1 or I² ≥ 50%). Publication bias was assessed using funnel plots and Egger’s test. Furthermore, meta-regression was used to examine the influence of potential moderators, including intervention, depression subtype/type, stimulation intensity, sex ratio, mean age, duration of illness, and treatment course, restricted to studies that reported these variables. The regression coefficient (β) was used to quantify the moderating effect.

For the network meta-analysis, a consistency random-effects model within a frequentist framework was employed, in which the basic parameters were estimated by generalised least squares and the between-study variance (τ²) by restricted maximum likelihood (REML), to synthesise both direct and indirect evidence across all interventions (29, 30). Indirect comparisons rely on the transitivity assumption: the unknown difference between interventions A and B is inferred from the known differences between A and C and between B and C, where C represents a common comparator. First, the networkplot command was used to generate a network graph depicting the quantitative relationships among studies and interventions. In the network plot, each node represents a distinct intervention and each edge represents a pairwise comparison between interventions; node size is scaled proportionally to the corresponding sample size. Second, global inconsistency between direct and indirect evidence was assessed using the mvmeta inconsistency command. For network structures forming closed loops, the network sidesplit all, tau command was applied to perform node-splitting analysis and further evaluate local inconsistencies. Subsequently, a random-effects model was fitted to synthesise the data and compare the relative efficacy of different electrical stimulation modalities. Finally, the sucra prob* command was used to rank the interventions and generate cumulative ranking probability plots. The surface under the cumulative ranking curve (SUCRA) quantifies the relative ranking of each intervention, with higher SUCRA values indicating greater efficacy.

## 3 Results

### 3.1 Research identification and selection

In accordance with the predefined search strategy, a total of 8,594 records were identified. Following automated duplicate detection in EndNote and manual verification, 3,340 duplicate records were removed, leaving 5,254 records for further screening. Titles and abstracts were then screened against the eligibility criteria, resulting in the exclusion of 4,962 records. The full texts of the remaining 292 articles were assessed in detail, of which 259 were excluded. Ultimately, 33 studies (13–17, 20, 31–57) met the inclusion criteria and were included in the NMA (**Figure 1**). A list of the included studies is provided in **Supplementary Digital Content 3**.

**Figure 1:**
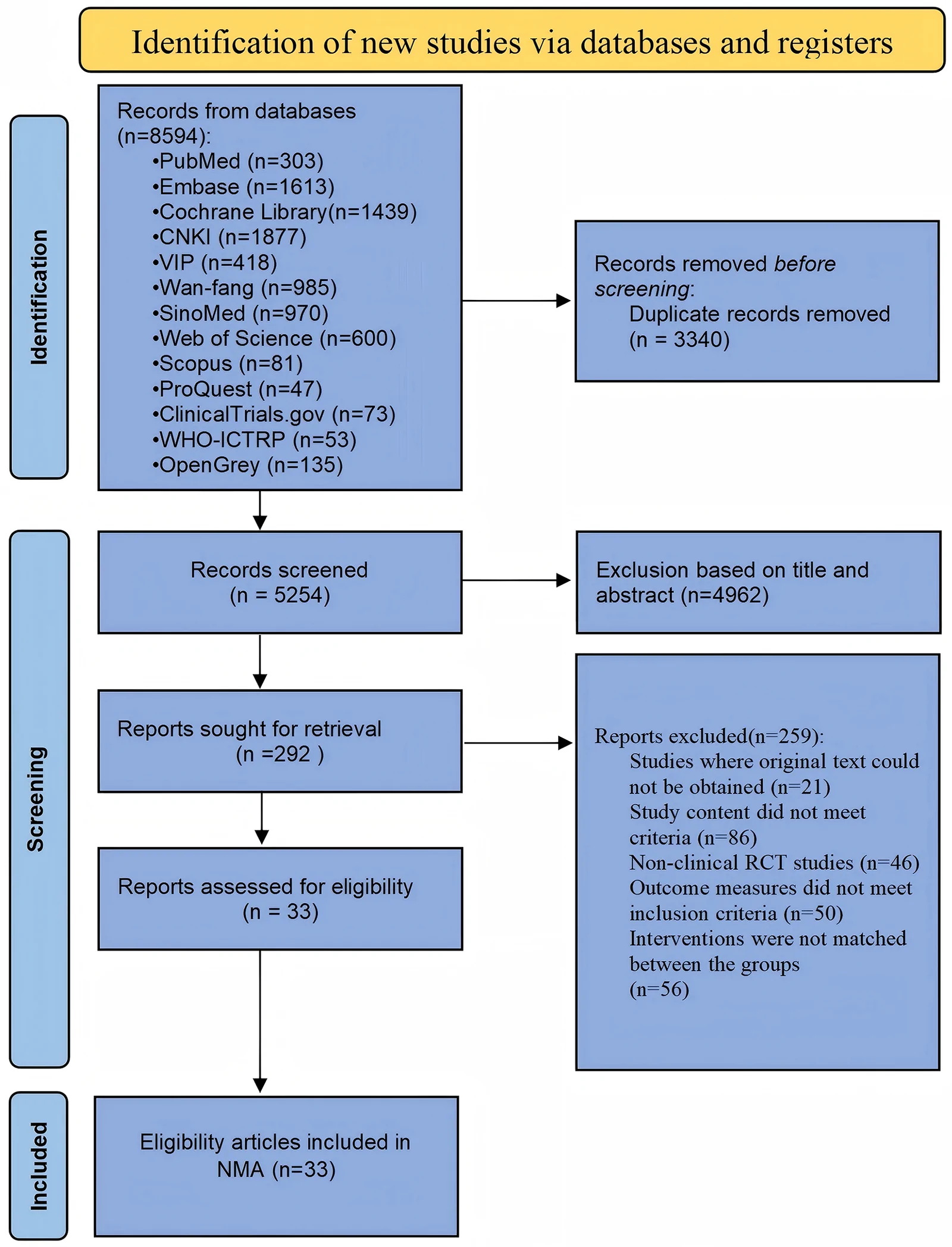
Flow diagram of study selection according to the PRISMA 2020 statement.

### 3.2 Characteristics of the included studies

The included studies comprised 4 multi-arm RCTs and 29 two-arm RCTs published between 2002 and 2026, encompassing 2,664 participants, 65.0% of whom were women. The interventions evaluated were high-dose unilateral ECT (HUL ECT), cranial electrotherapy stimulation (CES), bilateral frontal ECT (BF ECT), bilateral temporal ECT (BT ECT), low-dose unilateral ECT (LUL ECT), tACS, tDCS, VNS, taVNS, and TEAS. All control arms received sham electrical stimulation. Of the included studies, 11 reported the clinical response rate, 30 reported the HAMD score, and 7 reported adverse events. Study characteristics and electrical stimulation parameters are summarised in **Table 1** and **Supplementary Digital Content 4**.

**Table 1:** Characteristics of included studies.

Trials	Study design	Intervention		Patients number		Gender (male/female)		Age(years)		Duration of illness(Month)		Course of Treatment	Random Methods	Outcome
		Group 1	Group 2	Group1	Group2	Group1	Group2	Group1	Group2	Group1	Group2			
Song Zhiwen, 2010 (36)	RCT	HUL ECT	BF ECT	50	50	20/30	22/28	29.00 ± 16.00	31.00 ± 15.00	23.00 ± 17.00M	31.00 ± 15.00M	2W	Random	①
Luo Jiong, 2022 (47)	RCT	sham	tACS	29	28	7/22	3/25	42.97 ± 13.88	39.11 ± 15.73	70.45 ± 111.58d	67.57 ± 78.60d	4W	Random	②,③
Kong Suli, 2021 (46)	RCT	sham	CES	43	43	24/19	25/18	31.90 ± 8.70	31.50 ± 8.30	12.40 ± 3.20M	12.80 ± 3.70M	8W	Random Number Table Method	②
Gao Cunyou, 2021 (44)	RCT	sham	CES	34	33	18/16	18/15	31.70 ± 9.30	32.10 ± 8.90	12.60 ± 3.70M	12.90 ± 9.30M	8W	Random	②
Huang Chunhui, 2023 (49)	RCT	sham	tDCS	32	32	15/14	14/16	28.71 ± 5.98	29.86 ± 6.48	4.45 ± 2.27Y	4.24 ± 2.12Y	4W	Random Number Table Method	①,②,③
Li Qiong, 2022 (48)	RCT	sham	tDCS	40	40	17/23	19/21	68.20 ± 3.00	68.50 ± 2.88	5.85 ± 1.20Y	5.80 ± 1.20Y	4W	Random	①,②
Zhao Xin, 2024 (52)	RCT	LUL ECT	BT ECT	27	29	12/15	20/9	29.75 ± 13.23	32.79 ± 13.09	78.96 ± 107.36M	65.65 ± 76.35M	3W	Random Number Table Method	①,②,③
Tan Pei, 2024 (51)	RCT	HUL ECT	BT ECT	35	35	7/28	12/23	20.00 ± 11.11	22.00 ± 11.85	13.74 ± 7.86M	13.71 ± 10.2M	3W	Random	②,③
Li Wei, 2020 (43)	RCT	BT ECT	HUL ECT	36	36	15/21	12/24	39.50 ± 14.30	39.40 ± 14.50	138.32 ± 127.10d	76.00 ± 79.90d	4W	Random Number Table Method	②,③
		LUL ECT		36		17/19		34.70 ± 15.50		67.7 ± 87.0M		
Monika Dominiak, 2021 (45)	RCT	HUL ECT	BT ECT	45	46	23/23	21/24	57.06 ± 15.67	57.06 ± 15.67	16.00 ± 15.55M	18.00 ± 14.81M	12W	Random Number Table Method	①,②,③
Elham, Sharafi 2019 (41)	RCT	sham	tDCS	15	15	5/10	9/5	43.80 ± 12.60	50.70 ± 10.70	/		4W	Random Number Table Method	②
Peijing, Rong 2016 (39)	RCT	sham	taVNS	91	69	23/68	20/49	43.88 ± 13.95	40.10 ± 16.15	/		8W	Random Number Table Method	①,②
Charles H.Kellner, 2010 (34)	RCT	BF ECT	BT ECT	81	72	35/44	20/52	52.70 ± 14.70	51.70 ± 15.00	/		3W	Random Number Table Method	②
		HUL ECT		77		27/50		54.90 ± 15.30				
U. Palm, 2012 (37)	RCT	sham	tDCS	11	11	5/6	3/8	56.60 ± 12.00	58.60 ± 12.00	7.00 ± 10.00M	9.00 ± 15.00M	2W	Random Number Table Method	②
Pertti Heikman, 2002 (31)	RCT	BF ECT	HUL ECT	7	8	3/4	6/2	55.00 ± 14.81	61.00 ± 21.48	22.00 ± 71.11M	54.00 ± 66.66M	4W	Random Number Table Method	②
		LUL ECT		7		4/3		55.00 ± 19.25		20.00 ± 73.33M		
Rui Liu, 2026 (55)	RCT	sham	tACS	15	10	2/8	3/12	28.67 ± 5.97	31.20 ± 9.91	/		4W	Random Number Table Method	②
Jingjing, Zhou 2024 (17)	RCT	sham	tACS	33	33	8/25	6/27	27.48 ± 7.58	29.36 ± 8.76	49.00 ± 69.62d	54.00 ± 54.04d	4W	Random Number Table Method	①,②
Paulo S. Boggio, 2008 (33)	RCT	sham	tDCS	21	10	7/14	3/6	46.30 ± 5.80	51.60 ± 7.70	8.30 ± 7.60M	7.10 ± 6.20M	4W	Random Number Table Method	②
Ruonan Du, 2026 (53)	RCT	LUL ECT	HUL ECT	76	74	26/46	21/50	15.51 ± 1.27	15.55 ± 1.44	9.07 ± 1.40M	9.18 ± 1.62M	6W	Random Number Table Method	②
Yunhao Wu, 2026 (57)	RCT	sham	tDCS	60	60	22/38	29/31	35.72 ± 14.45	40.72 ± 16.62	4.40 ± 4.91M	6.08 ± 7.06M	4W	Random Number Table Method	①,②,③
Lucia Bulubas, 2019 (40)	RCT	sham	tDCS	16	15	3/13	7/8	42.00 ± 13.40	43.80 ± 11.20	/		6W	Random Number Table Method	②
Farzad Ranjkesh, 2007 (32)	RCT	BF ECT	BT ECT	14	13	6/8	5/8	33.40 ± 14.50	36.70 ± 7.50	/		8W	Random Number Table Method	②
		HUL ECT		12		4/18		34.10 ± 9.90				
Lucas Borrione, 2024 (13)	RCT	sham	tDCS	73	73	5/68	15/58	39.20 ± 8.80	38.80 ± 9.10	/		4W	Random Number Table Method	①,②
Hongxing Wang, 2022 (16)	RCT	sham	tACS	50	50	15/35	11/39	41.60 ± 12.80	38.30 ± 12.20	8.50 ± 4.30M	9.60 ± 3.80M	4W	Random Number Table Method	①,②
Tiejun Kang, 2026 (54)	RCT	sham	tACS	30	30	7/23	5/25	13.97 ± 1.19	14.47 ± 1.01	/		4W	Random Number Table Method	②
Ye Quan, 2026 (20)	RCT	sham	TEAS	30	30	5/25	5/25	23.50 ± 2.62	22.60 ± 2.28	/		4W	Random Number Table Method	②
Ting Zhang, 2026 (15)	RCT	sham	tDCS	26	31	6/20	6/25	28.58 ± 8.53	27.87 ± 9.53	24.00 ± 66.00M	2.00 ± 21.00M	8W	Random Number Table Method	②
D. Bennabi, 2015 (38)	RCT	sham	tDCS	12	12	7/5	2/10	60.4 ± 12.00	60.4 ± 12.00	/		4W	Random Number Table Method	②
Colleen K. Loo, 2010 (35)	RCT	sham	tDCS	20	20	9/11	9/11	45.60 ± 12.45	48.95 ± 10.00	21.55 ± 19.78M	15.85 ± 12.25M	4W	Random Number Table Method	②
Dan Li, 2024 (50)	RCT	sham	tDCS	25	31	4/21	7/24	28.48 ± 8.35	29.35 ± 8.71	/		4W	Random Number Table Method	②
Rachel D.Woodham, 2025 (14)	RCT	sham	tDCS	87	87	21/66	33/54	38.32 ± 10.92	37.09 ± 11.14	/		4W	Random Number Table Method	①
Tobias Schwippel, 2026 (56)	RCT	sham	tACS	10	10	2/8	2/8	39.60 ± 20.55	39.60 ± 20.55	/		2W	Random Number Table Method	②
John Rush, 2005 (54)	RCT	sham	VNS	110	112	44/66	51/59	45.90 ± 9.00	47.00 ± 9.00	46.60 ± 51.30M	46.60 ± 51.30M	10W	Random Number Table Method	①

RCT, Randomized Controlled Trial; sham: sham stimulation; HUL ECT, high-dose unilateral ECT; CES, cranial microcurrent stimulation; BF ECT, bilateral frontal lobe ECT; tACS, Transcranial Alternating Current Stimulation; BT ECT, Bilateral Temporal Lobe ECT; tDCS, Transcranial Direct Current Stimulation; LUL ECT, Low-Dose Unilateral ECT; VNS, Vagus Nerve Stimulation; TEAS, Transcutaneous Acupoint Electrical Stimulation; W, Week; M, Month; d, Day; ①: Clinical Response Rate; ②: HAMD Score; ③: Safety.

### 3.3 Risk of bias and certainty of evidence

**Figure 2** presents a summary of the risk-of-bias assessment; detailed assessments for individual studies are provided in **Supplementary Digital Content 5**. Twenty-eight studies were judged to be at low risk of bias, while five studies were rated as raising some concerns in the domains of the randomisation process and deviations from the intended intervention. The overall methodological quality was deemed acceptable.

**Figure 2:**
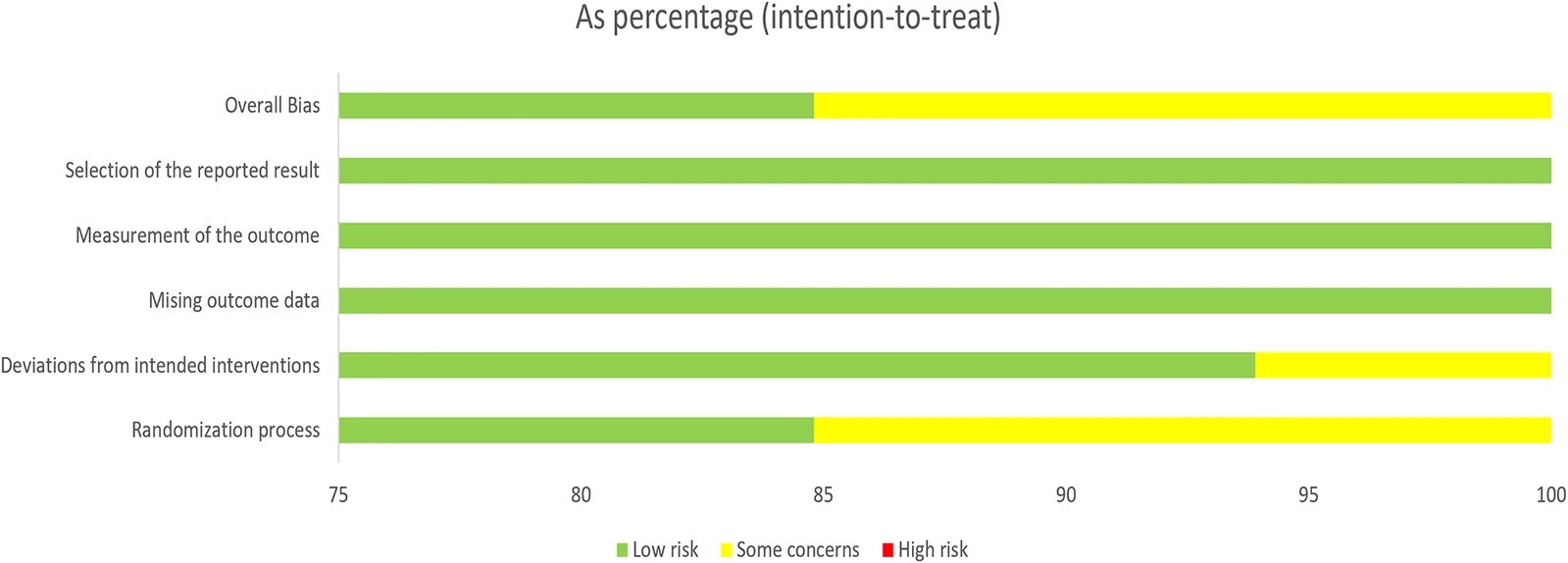
Risk of bias assessment summary.

The GRADE framework was applied to systematically evaluate the certainty of evidence for each outcome. The certainty of evidence was rated as low to moderate for the majority of comparisons, with methodological limitations in the included studies and imprecision in the effect estimates representing the primary reasons for downgrading. Detailed GRADE ratings for all comparisons are available in **Supplementary Digital Content 6**.

### 3.4 Effect-modifying characteristics

This study considered that potential differences in the distribution of covariates across intervention groups might affect the reliability of indirect comparisons. We therefore further examined the transitivity assumption by comparing potential effect modifiers across the intervention groups. Key baseline variables—including intervention, depression subtype/type, stimulation intensity, sex ratio, mean age, duration of illness, and treatment course—were similar across the 10 intervention types, with no obvious systematic differences (**Supplementary Digital Content 7**). This balance supports the transitivity assumption and indicates the validity of the unadjusted NMA.

### 3.5 Traditional meta-analysis results

A total of 12 RCTs including 1,368 participants (691 receiving sham electrical stimulation and 677 receiving electrical stimulation) reported the clinical response rate. Given substantial heterogeneity across studies (I² = 79%, P < 0.01), a random-effects model was applied. The meta-analysis (**Figure 3**) showed that, compared with sham stimulation, electrical stimulation was associated with a significantly higher clinical response rate in patients with depression (OR = 2.78, 95% CI 1.50 to 5.16).

**Figure 3:**
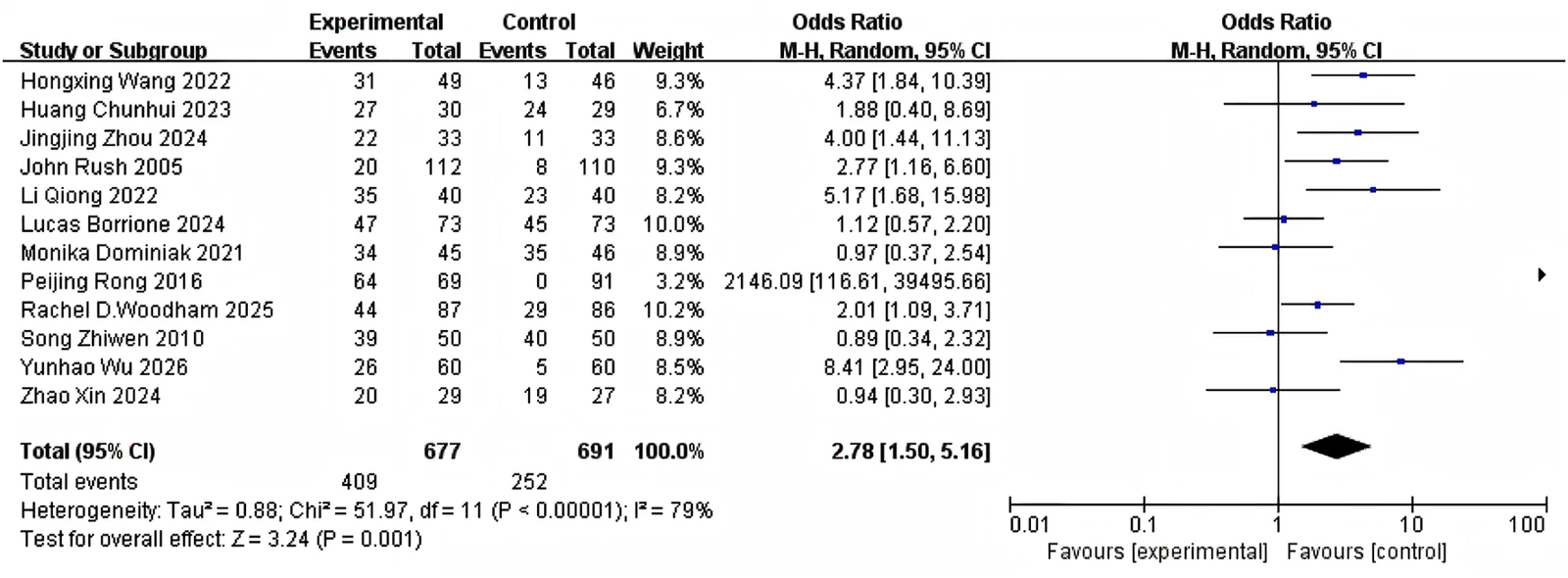
Forest plot of Clinical Response Rate for patients with depression treated with electrical stimulation.

A total of 30 RCTs including 1,958 participants (987 sham, 971 electrical stimulation) reported HAMD scores. A random-effects model was used owing to substantial heterogeneity (I² = 93%, P < 0.01). The meta-analysis (**Figure 4**) showed that electrical stimulation was associated with a significant reduction in depressive symptom severity, as measured by the HAMD score, compared with sham stimulation (SMD = −0.90, 95% CI −1.27 to −0.54).

**Figure 4:**
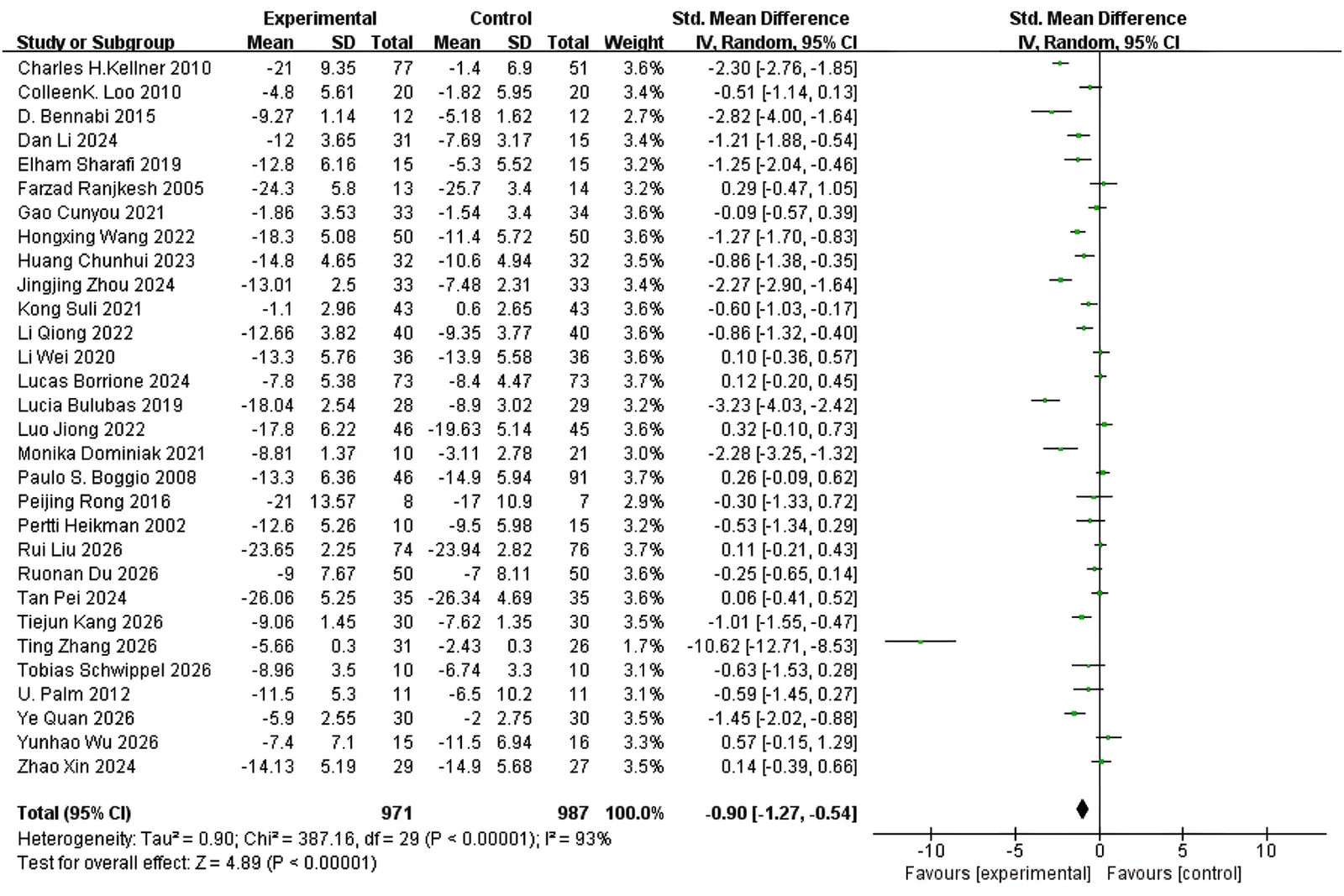
Forest plot of HAMD scores in patients with depression treated with electrical stimulation.

A total of 7 RCTs including 452 participants (225 sham, 227 electrical stimulation) reported safety outcomes. Heterogeneity was low (I² = 1%, P = 0.41); a fixed-effects model was therefore used. The meta-analysis (**Figure 5**) found no statistically significant difference in adverse events between electrical stimulation and sham stimulation (OR = 0.88, 95% CI 0.52 to 1.49).

**Figure 5:**
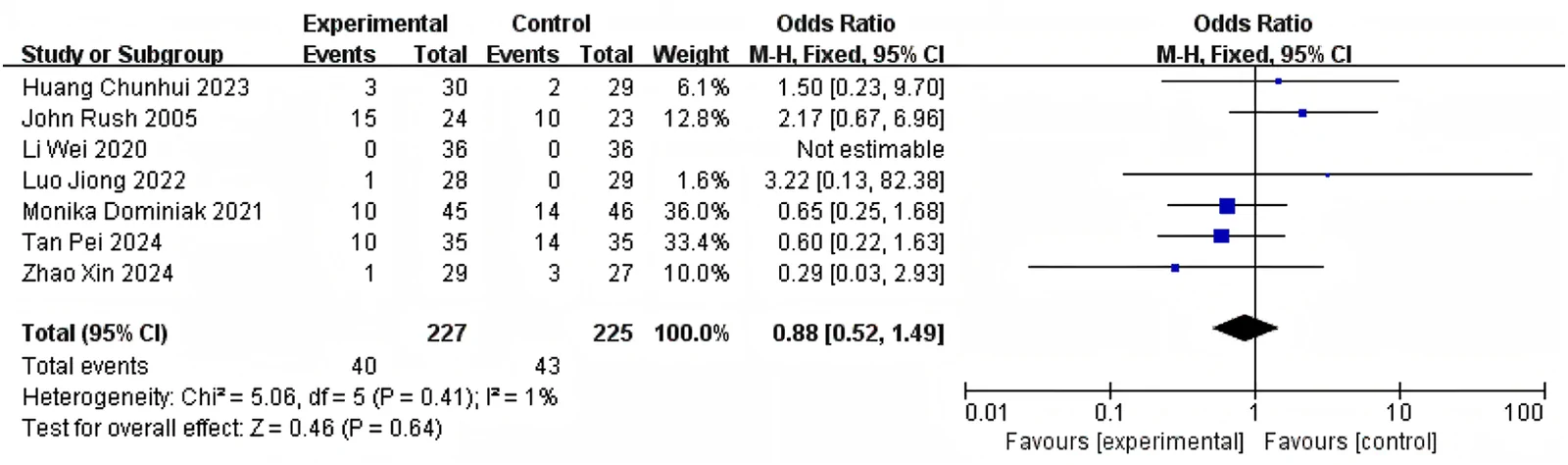
Forest plot of safety in patients with depression treated with electrical stimulation.

### 3.6 Network meta-analysis

#### 3.6.1 Clinical response rate

The NMA of the clinical response rate included 12 RCTs covering ninet interventions: BT ECT, HUL ECT, BF ECT, tDCS, LUL ECT, VNS, taVNS, tACS, and sham electrical stimulation. tDCS and sham stimulation were the most frequently evaluated interventions. The global heterogeneity test yielded a P value of 0.9453 (> 0.05), indicating no significant heterogeneity across the network. As the evidence network contained no closed loops, a consistency model was fitted. **Figure 6A** provides an overview of the evidence network.

**Figure 6:**
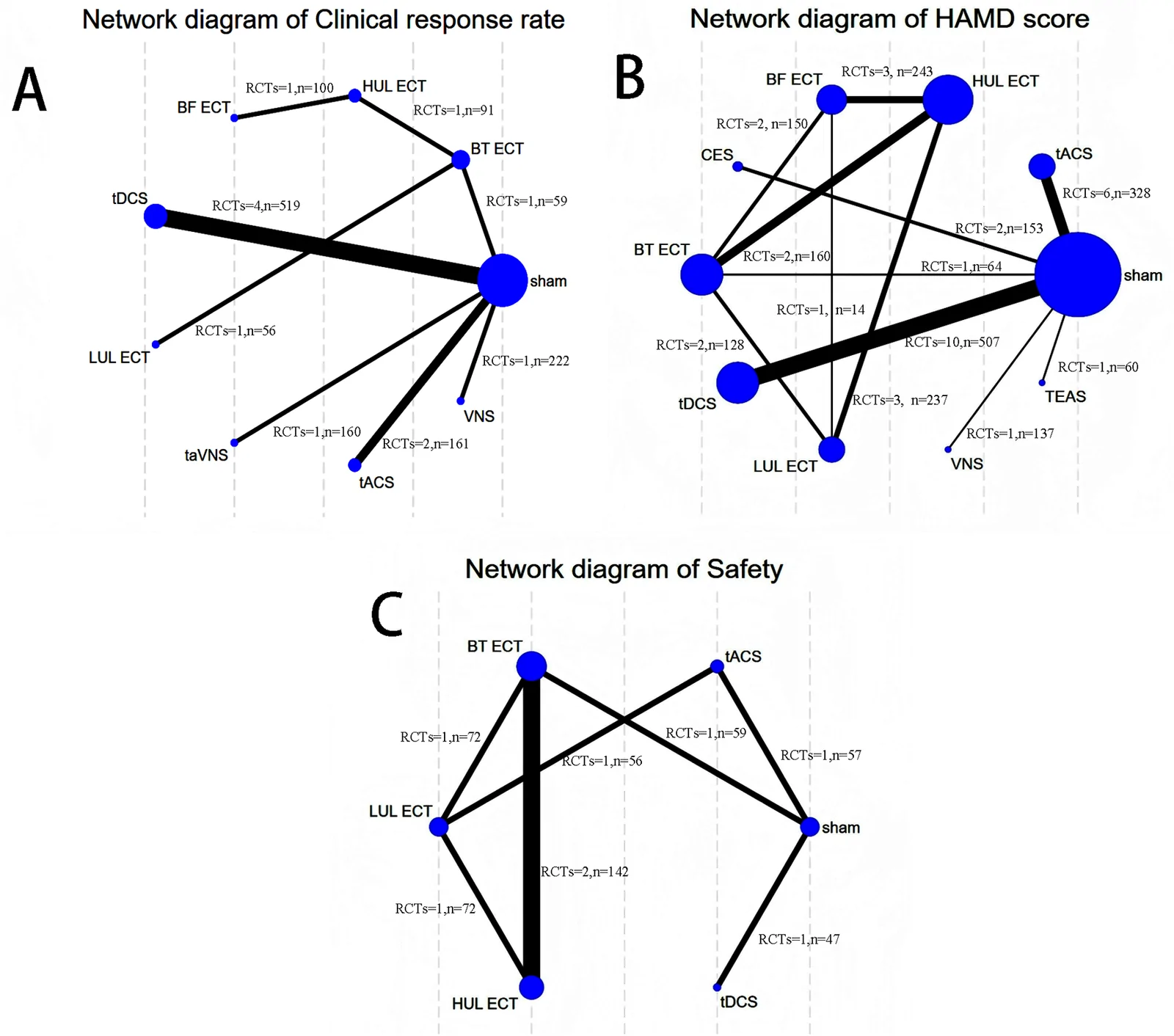
Network diagram of intervention comparisons. **(A)** Network diagram of clinical response rate. **(B)** Network diagram of HAMD score. **(C)** Network diagram of safety.

The NMA results (**Table 2**) showed that compared with sham electrical stimulation, tDCS (OR = 1.59, 95% CI 1.00 to 2.52), tACS (OR = 2.12, 95% CI 1.15 to 3.34), and taVNS (OR = 4.12, 95% CI 3.08 to 5.16) were associated with higher clinical response rates. Compared with tACS, taVNS (OR = 3.26, 95% CI 1.72 to 4.80) was associated with a higher clinical response rate.

**Table 2:** Network meta-analysis of Clinical Response Rate.

	BT ECT	HUL ECT	BF ECT	tDCS	LUL ECT	taVNS	tACS	VNS
BT ECT	−							
HUL ECT	0.99 (0.48,2.07)	−						
BF ECT	1.02 (0.36,2.85)	1.03 (0.50,2.11)	−					
tDCS	0.69 (0.29,1.62)	0.69 (0.22,2.13)	0.67 (0.18,2.57)	−				
LUL ECT	0.98 (0.45,2.13)	0.99 (0.34,2.87)	0.96 (0.27,3.49)	1.43 (0.45,4.55)	−			
taVNS	0.01 (0.00,0.12)	0.01 (0.00,0.13)	0.01 (0.00,0.14)	0.01 (0.00,0.17)	0.01 (0.00,0.14)	−		
tACS	0.51 (0.20,1.33)	0.52 (0.16,1.72)	0.50 (0.12,2.05)	0.75 (0.35,1.62)	0.52 (0.15,1.79)	3.26 (1.72, 4.80)	−	
VNS	0.44 (0.12,1.58)	0.45 (0.10,1.93)	0.43 (0.08,2.23)	0.65 (0.21,2.02)	0.45 (0.10,2.00)	2.12 (-4.08, 8.32)	0.86 (0.26,2.89)	−
sham	1.09 (0.53,2.24)	1.10 (0.39,3.07)	1.07 (0.30,3.76)	1.59 (1.00,2.52)	1.11 (0.38,3.21)	4.12 (3.08, 5.16)	2.12 (1.15,3.92)	2.46 (0.87,6.96)

The SUCRA values for the clinical response rate, ranked in descending order, were as follows:taVNS (99.9%) > VNS (70.0%) > tACS (67.3%) >tDCS (53.6%) > LUL ECT (34.4%) > HUL ECT (33.7%) > BF ECT (33.3%) > BT ECT (32.1%) > sham (25.6%). The corresponding SUCRA curves are displayed in **Figure 7A**.

**Figure 7:**
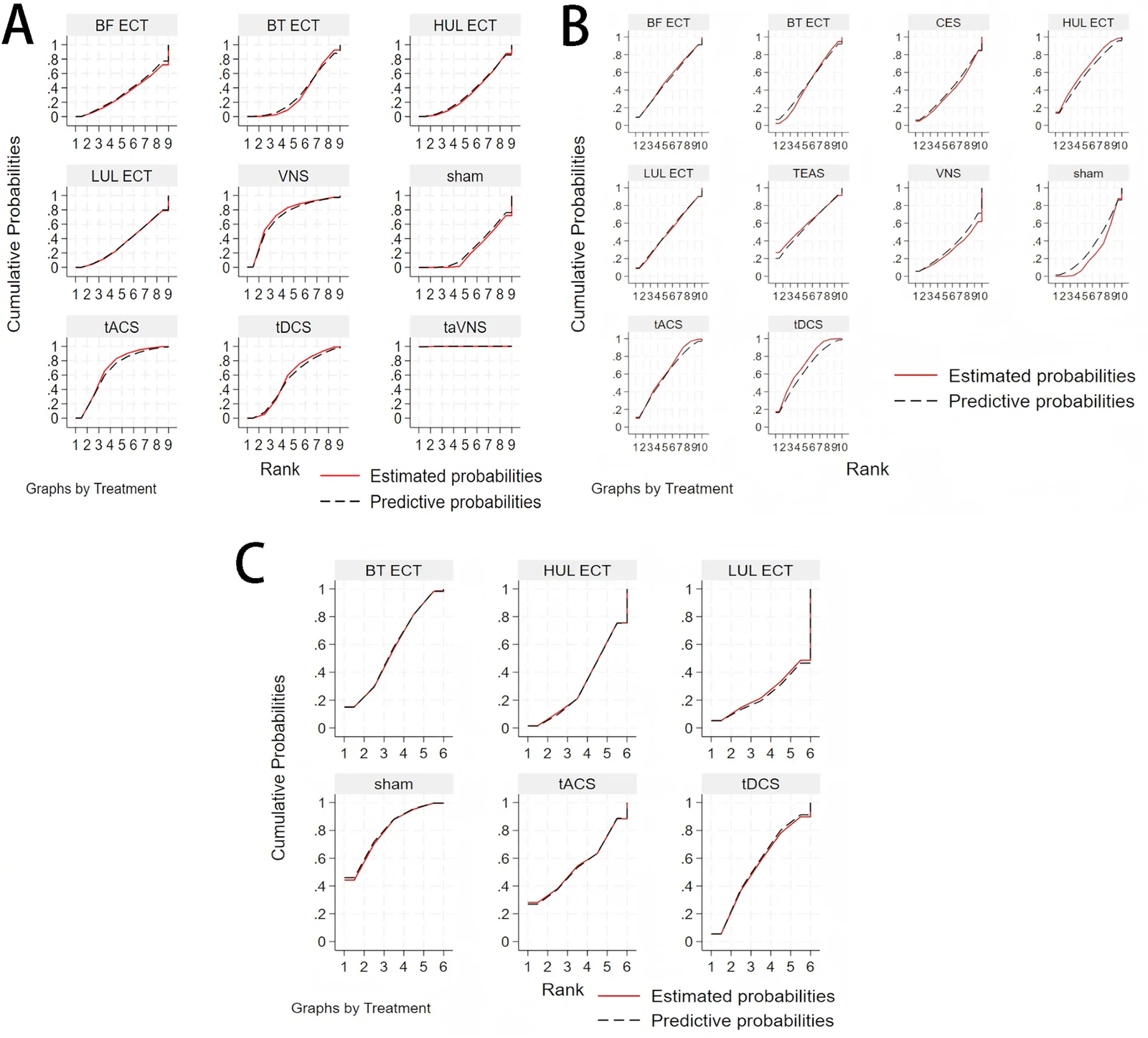
Cumulative rank-probability curves for **(A)** Clinical Response Rate, **(B)** HAMD Score, **(C)** Safety.

#### 3.6.2 HAMD score

The NMA of the HAMD score included 30 RCTs covering ten interventions: tACS, HUL ECT, BF ECT, CES, BT ECT, tDCS, LUL ECT, VNS, TEAS, and sham electrical stimulation, with tDCS and sham being the most frequently reported comparators. The global heterogeneity test was non-significant (P = 0.9987, > 0.05). Four closed loops were identified and assessed for local inconsistency: the inconsistency factor (IF) for the HUL ECT–BF ECT–LUL ECT loop was 0.670 (95% CI 0.00 to 3.93); for the HUL ECT–BF ECT–BT ECT loop, 0.624 (95% CI 0.00 to 2.69); for the HUL ECT–BT ECT–LUL ECT loop, 0.573 (95% CI 0.00 to 2.60); and for the BF ECT–BT ECT–LUL ECT loop, 0.021 (95% CI 0.00 to 1.30). All 95% CIs encompassed zero, indicating no evidence of inconsistency (**Figure 8A**). Accordingly, a consistency model was used; the evidence network is presented in **Figure 6B**.

**Figure 8:**
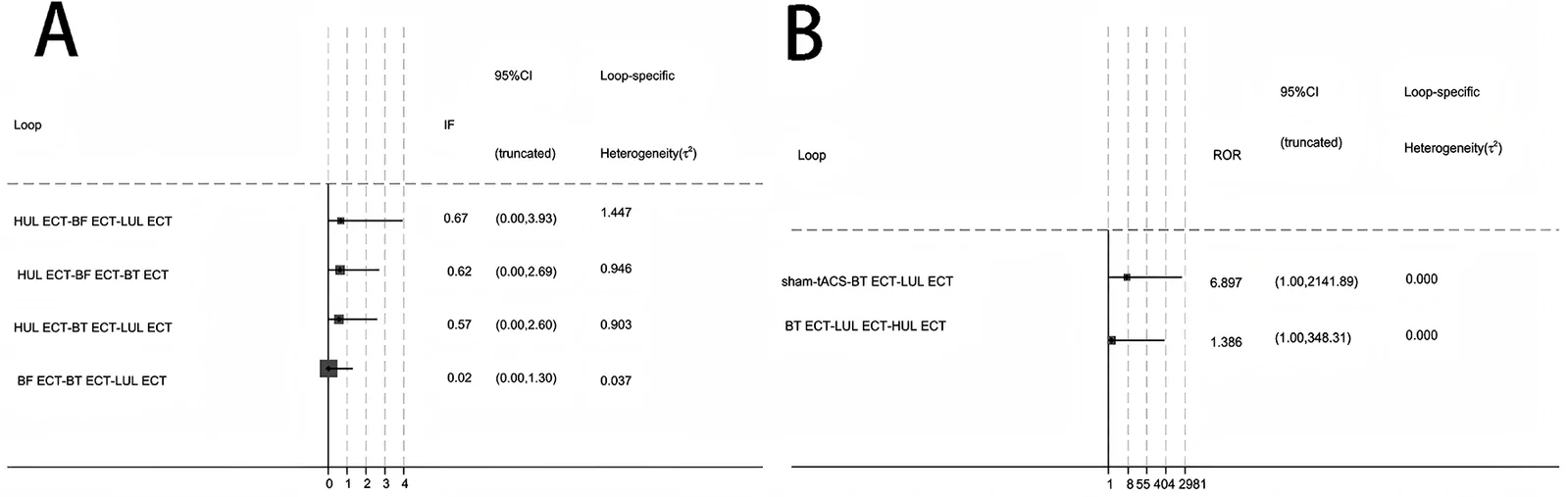
**(A)** HAMD Score inconsistency testing; **(B)** Safety inconsistency testing.

The NMA results (**Table 3**) indicate that, compared with sham electrical stimulation, tDCS (SMD = −1.70, 95% CI −2.80 to −0.60) was associated with a greater reduction in HAMD scores.

**Table 3:** Network meta-analysis of HAMD scores.

	tACS	HUL ECT	BF ECT	CES	BT ECT	tDCS	LUL ECT	VNS	TEAS
tACS	−								
HUL ECT	0.02 (-3.87,3.91)	−							
BF ECT	-0.36 (-4.42,3.70)	-0.38 (-1.98,1.22)	−						
CES	-1.00 (-3.75,1.75)	-1.02 (-5.37,3.32)	-0.65 (-5.14,3.85)	−					
BT ECT	-0.49 (-4.13,3.15)	-0.51 (-1.89,0.88)	-0.13 (-1.93,1.67)	0.52 (-3.60,4.63)	−				
tDCS	0.36 (-1.42,2.13)	0.33 (-3.47,4.13)	0.71 (-3.26,4.68)	1.36 (-1.26,3.97)	0.84 (-2.70,4.38)	−			
LUL ECT	-0.45 (-4.50,3.60)	-0.47 (-2.15,1.21)	-0.09 (-2.15,1.97)	0.55 (-3.93,5.04)	0.04 (-1.75,1.82)	-0.80 (-4.77,3.16)	−		
VNS	-1.61 (-5.23,2.01)	-1.63 (-6.57,3.31)	-1.25 (-6.32,3.82)	-0.61 (-4.71,3.49)	-1.12 (-5.86,3.62)	-1.97 (-5.48,1.55)	-1.16 (-6.23,3.91)	−	
TEAS	0.10 (-3.55,3.75)	0.08 (-4.88,5.04)	0.46 (-4.63,5.55)	1.10 (-3.02,5.23)	0.59 (-4.17,5.35)	-0.25 (-3.80,3.29)	0.55 (-4.54,5.64)	1.71 (-3.04,6.46)	−
sham	-1.35 (-2.74,0.04)	-1.37 (-5.01,2.27)	-0.99 (-4.80,2.82)	-0.35 (-2.72,2.03)	-0.86 (-4.22,2.50)	-1.70 (-2.80,-0.60)	-0.90 (-4.71,2.91)	0.26 (-3.08,3.60)	-1.45 (-4.82,1.92)

The SUCRA values for the HAMD score, ranked in descending order, were as follows: tDCS (71.3%) > HUL ECT (64.0%) > tACS (62.3%) > TEAS (60.2%) > BF ECT (52.1%) > LUL ECT (49.6%) > BT ECT (47.4%) > CES (38.2%) > VNS (28.9%) > sham (26.0%). The corresponding SUCRA curves are presented in **Figure 7B**.

#### 3.6.3 Safety

The NMA of safety outcomes included 7 RCTs covering six interventions: tACS, BT ECT, LUL ECT, HUL ECT, tDCS, and sham electrical stimulation, with BT ECT and HUL ECT being the most frequently reported modalities. The evidence network formed two triangular closed loops. The global inconsistency test was non-significant (P = 0.8029, > 0.05). Local inconsistency assessment yielded a ratio of odds ratios (ROR) of 6.897 (95% CI 1.00 to 2,141.89) for the sham–tACS–BT ECT–LUL ECT loop and an ROR of 1.386 (95% CI 1.00 to 348.31) for the BT ECT–LUL ECT–HUL ECT loop. As all 95% CIs included 1, no significant inconsistency was detected (**Figure 8B**). A consistency model was therefore adopted; the evidence network is shown in **Figure 6C**.

The NMA results (**Table 4**) revealed no statistically significant differences among the interventions, with all 95% CIs including 1, suggesting that none of the five active electrical stimulation modalities were associated with a significantly elevated risk of adverse events relative to sham stimulation.

**Table 4:** Network meta-analysis of safety.

	tACS	BT ECT	LUL ECT	HUL ECT	tDCS
tACS	—				
BT ECT	0.98 (0.07,14.31)	—			
LUL ECT	0.42 (0.06,3.11)	0.43 (0.03,5.95)	—		
HUL ECT	0.70 (0.05,10.55)	0.72 (0.45,1.16)	1.69 (0.12,24.03)	—	
tDCS	1.18 (0.09,16.24)	1.21 (0.22,6.73)	2.83 (0.18,44.66)	1.67 (0.28,9.91)	—
sham	1.69 (0.13,21.98)	1.73 (0.34,8.81)	4.07 (0.27,60.62)	2.41 (0.44,13.01)	1.44 (0.82,2.52)

The SUCRA values for safety, ranked in descending order, were as follows: sham (80.3%) > BT ECT (56.6%) > tDCS (54.9%) > tACS (54.0%) > HUL ECT (31.1%) > LUL ECT (23.1%). The corresponding SUCRA curves are presented in **Figure 7C**.

### 3.7 Publication bias and sensitivity analysis

Funnel plots were generated to assess publication bias for the clinical response rate, HAMD score, and safety outcomes. Although the majority of data points were symmetrically distributed around the central reference line within the 95% confidence limits, a subset of studies fell outside these boundaries, yielding an asymmetric distribution suggestive of possible publication bias. Funnel plots for each outcome are presented in **Figure 9**.

**Figure 9:**
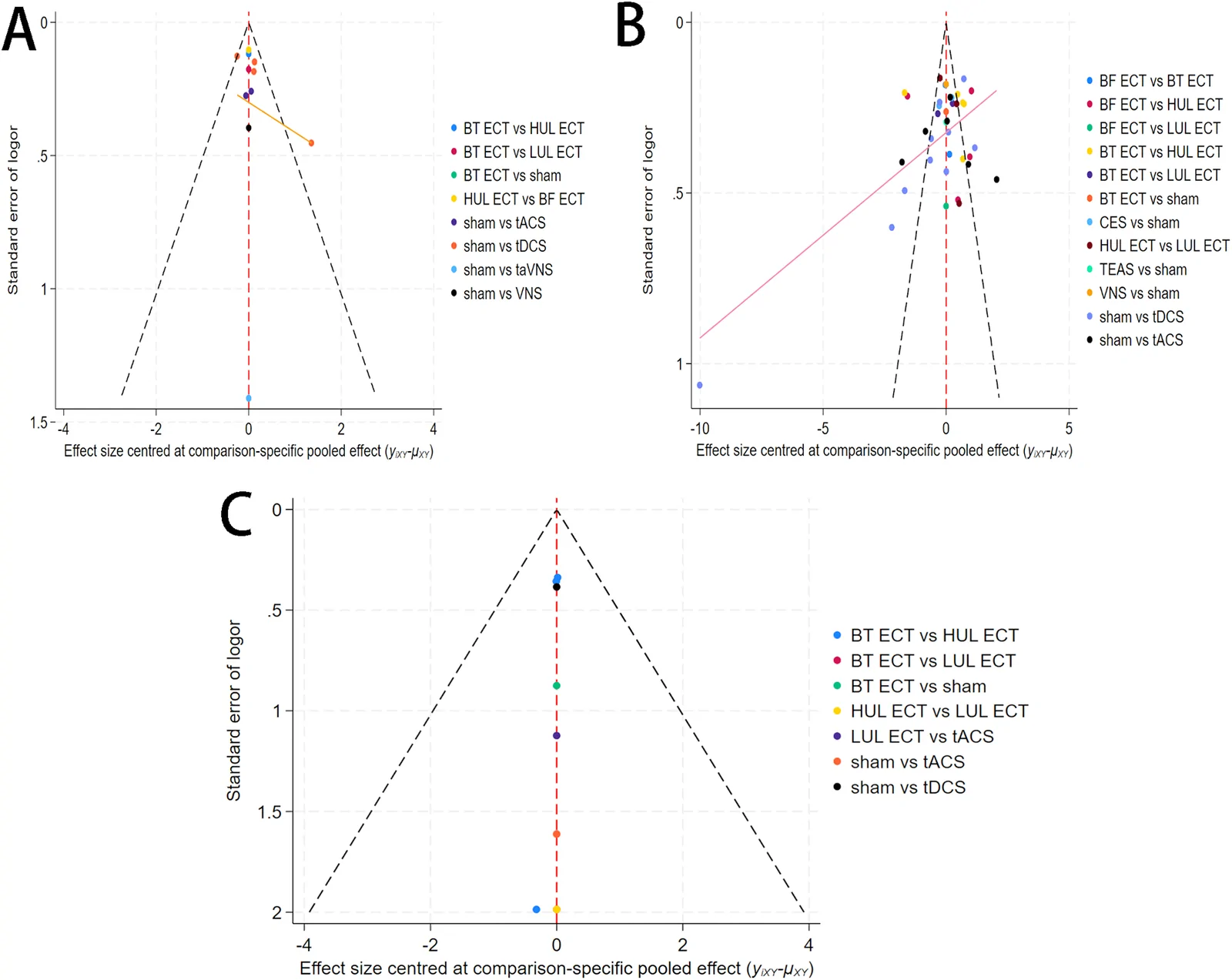
**(A)** Funnel plot of Clinical Response Rate, **(B)** Funnel plot of HAMD Score, **(C)** Funnel plot of Safety.

Egger’s test was performed to further evaluate funnel plot asymmetry (**Table 5**). No statistically significant evidence of publication bias was detected for the clinical response rate (P = 0.933), the HAMD score (P = 0.057), or safety outcomes (P = 0.709).

**Table 5:** Egger’s test was performed on the outcome measures reported in this study to assess publication bias.

Outcome indicators	Egger(P-value)	
Clinical Response Rate	0.933	>0.05
HAMD Score	0.057	>0.05
Safety	0.709	>0.05

Sensitivity analyses were conducted for the primary outcomes to explore potential sources of heterogeneity. The results remained materially unchanged (see **Supplementary Digital Content 8** for details), indicating that the findings are robust.

### 3.8 Meta-regression

We conducted meta-regression analyses to assess whether interventions, depression subtype/type, stimulation intensity, gender ratio, mean age, course of disease, and treatment course affected the pooled estimates of clinical response rate and HAMD scores. The 95% confidence intervals of all covariates crossed zero, and all P values exceeded 0.05, suggesting no statistically significant associations (**Table 6**).

**Table 6:** The results of univariate meta-regression analysis.

Potential moderators	Regression coefficient,β (95% CI)	Inclusion of zero within the 95% CI of β	P-value	Is the p-value greater than 0.05?
Clinical response rate				
Interventions	16.72 (-36.77, 70.20)	Y	0.435	Y
Depression subtype/type	19.91 (-141.54, 181.36)	Y	0.749	Y
Intensity	−0.48 (−7.85, 6.89)	Y	0.865	Y
Gender Ratio	23.56 (−151.15, 198.27)	Y	0.727	Y
Age	0.46 (−4.53, 5.45)	Y	0.810	Y
Course of Disease	6.53 (−406.59, 419.65)	Y	0.390	Y
Treatment Course	−4.56 (−22.64, 13.52)	Y	0.522	Y
HAMD scores				
Interventions	0.01 (−0.43, 0.45)	Y	0.964	Y
Depression subtype/type	1.17 (−0.63, 2.97)	Y	0.190	Y
Intensity	0.002 (−0.004, 0.008)	Y	0.433	Y
Gender Ratio	1.54 (−0.89, 3.97)	Y	0.203	Y
Age	0.03 (−0.04, 0.09)	Y	0.399	Y
Course of Disease	0.0003 (−0.12, 0.12)	Y	0.996	Y
Treatment Course	−0.09 (−0.47, 0.28)	Y	0.605	Y

## 4 Discussion

### 4.1 Main finding

This systematic review and NMA evaluated the efficacy of nine electrical stimulation modalities for depression across 33 RCTs involving 2,664 patients. The findings indicate that electrical stimulation promotes clinical improvement in patients with depression, as reflected in both the clinical response rate and depressive symptom severity (HAMD score), and is associated with an acceptable safety profile. These results are concordant with those of previous studies (21–24). However, therapeutic effects varied across modalities. Specifically, compared with other forms of electrical stimulation and sham stimulation, taVNS demonstrated a relative advantage in improving the clinical response rate, whereas tDCS showed a relative advantage in reducing depressive symptom severity.

### 4.2 Interpretation

Electrical stimulation, as a core neuromodulation strategy for depression, has been shown to be superior to sham stimulation in improving clinical response rates and reducing depressive symptom severity, while maintaining a favourable safety profile. These findings corroborate and extend prior evidence from individual studies and pairwise meta-analyses. A recent meta-analysis of tDCS for treatment-resistant depression, for instance, reported consistent benefits of tDCS therapy (24).

taVNS attained the highest SUCRA value for the clinical response rate, indicating the highest estimated probability of ranking best for this outcome. However, SUCRA reflects ranking probability and does not constitute proof of clinical superiority, and the effect estimate was imprecise, with low certainty of evidence. The biological plausibility of this ranking is nonetheless supported by converging evidence from neuroimaging and biomarker studies: vagal afferent fibres project to the nucleus tractus solitarius, which in turn modulates the locus coeruleus noradrenergic system, the dorsal raphe nucleus serotonergic system, and limbic structures including the amygdala and hippocampus (58, 59). Furthermore, chronic, intermittent vagus nerve stimulation has been shown to induce sustained neuroplastic changes, including increased hippocampal brain-derived neurotrophic factor (BDNF) expression and enhanced neurogenesis, which may underlie the progressive and delayed therapeutic response characteristic of vagal stimulation (60, 61). This interpretation nonetheless warrants caution: the included evidence largely reflects non-invasive taVNS, whose typical stimulation parameters are a frequency of 20 Hz, an intensity of 4–6 mA, and a pulse width of 0.2 ms (39). Given the limited number of trials and the imprecision of the estimates, future studies with larger sample sizes, standardised stimulation parameters, and extended follow-up are warranted to refine effect estimates and identify the patient subgroups most likely to benefit from taVNS.

tDCS delivers low-intensity direct current via scalp electrodes to induce polarity-dependent, subthreshold modulation of cortical excitability. tDCS ranked highest for the HAMD score and was superior to sham stimulation in reducing depressive symptom severity. This finding aligns with a previous individual-participant data meta-analysis of nine tDCS trials, which confirmed a clinically meaningful reduction in depression severity scores with active stimulation relative to sham (12). Several factors may account for the strong performance of tDCS on this endpoint. First, tDCS is the most extensively studied modality in this network, contributing the largest pooled sample size and thus affording greater statistical precision for the HAMD score outcome. Second, tDCS targets the dorsolateral prefrontal cortex (DLPFC), a region critical to cognitive control and emotional regulation, and its mechanism of action involves NMDA receptor-dependent long-term potentiation-like plasticity (18). Recent mechanistic studies have confirmed that repeated tDCS sessions produce cumulative neuroplastic after-effects that persist beyond the stimulation period, a phenomenon mediated by BDNF secretion and calcium-dependent synaptic remodelling (62, 63). Third, several practical innovations—including remotely supervised, home-based tDCS protocols—have substantially reduced participant burden and enhanced ecological validity, enabling the inclusion of larger and more representative samples (13, 14). However, tDCS ranked appreciably lower for the clinical response rate than for the HAMD score. This discrepancy between categorical response and continuous symptom severity outcomes suggests that tDCS may produce moderate but broadly distributed symptom improvements across the study population, rather than driving a subset of patients to cross formal response thresholds. This pattern has been observed in previous tDCS trials, where effect sizes on continuous severity scales were more robust than those on response rate measures (64), and may reflect the relatively low stimulation intensity and short treatment duration employed in some of the included RCTs. Optimising stimulation parameters—including current intensity (e.g., increasing from 1–2 mA to 3–4 mA), session frequency (e.g., twice-daily regimens), total number of sessions, and individualised targeting using structural MRI-guided neuronavigation—may help enhance the capacity of tDCS to elicit a clinical response (15, 65).

ECT remains a highly effective acute treatment for severe and treatment-resistant depression. Treatment efficacy varies by electrode placement; HUL ECT ranked highest among ECT variants for the HAMD score, and its clinical response rate was comparable to that of bilateral electrode placements. This finding is consistent with recent evidence suggesting that optimisation of the unilateral ECT dose based on individual seizure thresholds can approach or match the efficacy of bilateral ECT while preserving advantages in cognitive tolerability (10, 23). In this network, bilateral temporal ECT performed relatively poorly on both efficacy outcomes; this finding warrants careful interpretation and may reflect indication bias—bilateral ECT tends to be prioritised for patients with more severe illness, psychotic features, catatonic features, or acute suicide risk, populations in whom achieving an adequate clinical response is inherently more challenging (34). Real-world and observational data have consistently documented that participants receiving bilateral ECT exhibit higher pre-treatment psychopathological severity than those receiving unilateral ECT; the selection bias thus introduced may have attenuated the apparent efficacy of bilateral ECT in uncorrected network comparisons (11). Importantly, across all four ECT variants, a meaningful hierarchy of clinical response rates was observed, confirming that ECT as a class remains among the most effective acute interventions for major depressive disorder—a conclusion consistent with the findings of a recent systematic review and meta-analysis spanning three decades of ECT efficacy research (23).

tACS demonstrated moderate efficacy on both the clinical response rate and the HAMD score, supporting its potential as a non-invasive, frequency-tuned neuromodulation approach. Recent EEG studies have lent support to the premise that tACS can entrain endogenous alpha oscillations (8–12 Hz). Alpha activity in the left frontal region is characteristically asymmetric in depression, and these studies confirm that 10-Hz tACS applied to the left DLPFC enhances alpha power and functional connectivity within fronto-limbic networks (16). However, the heterogeneity of stimulation parameters across the included tACS trials—which encompassed 10-Hz, 40-Hz, and individualised alpha-frequency protocols—limits the precision of the pooled effect estimates and underscores the need for parameter standardisation. CES and TEAS have been less extensively investigated in the controlled-trial literature; their efficacy rankings were moderate, and they did not achieve statistical significance in most pairwise comparisons. Given the limited number of eligible RCTs, their ranking values should be regarded as provisional. Further placebo-controlled trials with adequate sample sizes are required before definitive conclusions can be drawn regarding their role in the therapeutic armamentarium (22).

In RCTs reporting adverse event data, the incidence of adverse events was not significantly elevated for electrical stimulation relative to sham stimulation. The SUCRA ranking identified sham as the condition with the most favourable safety profile, followed by BT ECT and tDCS, with LUL ECT ranking lowest. However, safety outcomes were reported for only a subset of the interventions in the network, namely tACS, BT ECT, LUL ECT, HUL ECT, and tDCS. This represents a limitation of the present study, as the absence of safety data for the remaining interventions precluded a comprehensive comparative assessment of adverse events across all electrical stimulation modalities. Consequently, the safety-related findings and any associated ranking should be interpreted with caution, because they reflect only a subset of the included interventions and may not capture the full safety profile of electrical stimulation. Future randomised controlled trials should routinely report adverse events for every intervention, ideally using standardised and validated instruments, to enable more robust and complete comparative safety analyses.

### 4.3 Strengths and limitations

This study employed an NMA framework to systematically compare the therapeutic effects of different electrical stimulation modalities for depression, establishing, to our knowledge, the first efficacy ranking for nine commonly used electrical stimulation interventions. The use of sham electrical stimulation as the control condition helped preserve the rigour of blinded designs across the included trials. The findings not only provide evidence-based guidance for clinicians in selecting optimal treatment strategies but also help delineate priorities for future research, thereby contributing to the maximisation of patient benefit. The reliability of these conclusions rests on a systematic and comprehensive study design: the research team implemented a systematic search across 13 databases, synthesised data from 2,664 patients with depression, and maintained rigorous quality control throughout the review process to ensure the robustness of the results. Building on this foundation, evidence-based recommendations were formulated with explicit reference to the certainty of evidence, thereby enhancing the dual value of this study as a guide for both clinical practice and scientific enquiry.

Nevertheless, this NMA has several limitations that merit consideration. First, although 33 RCTs were included, the evidence network remains notably sparse. Specifically, the number of original studies supporting certain interventions (such as VNS and TEAS) is limited, and individual study sample sizes are generally modest, which may yield imprecise effect estimates. In addition, ECT was split into four independent nodes (BT, BF, HUL, and LUL), which increased the number of nodes in the network while reducing the number of studies available for each ECT variant; this greater network sparsity may have limited the statistical power and precision of the ECT subgroup estimates and increased their uncertainty, such that the effect estimates for individual ECT techniques should be interpreted with caution and confirmed in future studies with larger sample sizes. Second, stimulation parameters, including frequency, intensity, and pulse width, exert a decisive influence on neurophysiological outcomes; however, owing to limitations in the available primary data, this study was unable to account for fundamental differences in these parameters across trials. This limitation may have led to the artificial homogenisation of heterogeneous interventions, masking actual mechanistic-level variability and thereby blurring the true boundaries of efficacy among stimulation modalities, while increasing the uncertainty in the interpretation of pooled effect sizes. Third, certain analyses were constrained by potential heterogeneity in participant characteristics, including illness duration, treatment history, age, and depression severity. These factors may introduce confounding in the estimation of intervention effects, thereby reducing the precision and generalisability of the findings. Finally, the certainty of evidence was inconsistent across comparisons; several of the original studies lacked detailed reporting of random sequence generation and allocation concealment, weakening the overall credibility of the evidence base.

## 5 Conclusions

This NMA indicates that electrical stimulation is associated with improvements in the clinical response rate and depressive symptom severity in patients with depression, with an acceptable tolerability profile. Therapeutic effects vary across electrical stimulation modalities. Specifically, taVNS demonstrated a relative advantage in improving the clinical remission rate; however, this reflects ranking probability rather than established superiority, and its effect was accompanied by a wide confidence interval, whereas tDCS showed a relative advantage in alleviating depressive symptom severity. Nevertheless, given the generally low certainty of the existing evidence, these findings should be interpreted with caution. Further verification in larger-scale, higher-quality randomised controlled trials is warranted.

## Data Availability

The original contributions presented in the study are included in the article/**Supplementary Material**, further inquiries can be directed to the corresponding author/s.
