# Supplementary material for: Effects of different electrical stimulation on depression: a systematic review and network meta-analysis
Source: Front Psychiatry. 2026 Oct 27;17:1962608. doi: 10.3389/fpsyt.2026.1962608 (PMC13748327; doi:10.3389/fpsyt.2026.1962608)
Supplement: Supplementary file 1 [file DataSheet1.docx]

SUPPLEMENTARYDIGITALCONTENT

**[Supplementary Digital Content 1.](#_Toc11749)** [Checklist of Items to Include When Reporting a Systematic Review Involving a Network Meta-analysis.	1](#_Toc11749)

**[Supplementary Digital Content 2.](#_Toc17382)** [Details of the search strategy for the Chinese and English databases.	6](#_Toc17382)

**[Supplementary Digital Content 3.](#_Toc25636)** [The list of included articles.	8](#_Toc25636)

**[Supplementary Digital Content 4.](#_Toc8345)** [Electrical stimulation parameters and study designdesign	11](#_Toc8345)

**[Supplementary Digital Content 5.](#_Toc2012)** [Risk of bias assessment	17](#_Toc2012)

**[Supplementary Digital Content 6.](#_Toc24478)** [The GRADE assessment.	18](#_Toc24478)

**[Supplementary Digital Content 7.](#_Toc17771)** [Effect-modifying characteristics	21](#_Toc17771)

**[Supplementary Digital Content 8.](#_Toc19386)** [Sensitivity Analysis	22](#_Toc19386)

## Supplementary Digital Content 1. Checklist of Items to Include When Reporting a Systematic Review Involving a Network Meta-analysis.

| **Section/Topic** | **Item**  **No*** | **Checklist item†** | **Reported**  **on Page #** |
| --- | --- | --- | --- |
| **TITLE** |  |  |  |
| Title | 1 | Identify the report as a systematic review *incorporating a network meta-analysis (or related form of meta- analysis)*. | Title |
| **ABSTRACT** |  |  |  |
| Structured summary | 2 | Provide a structured summary including, as applicable: Background: main objectives  Methods: data sources; study eligibility criteria, participants, and interventions; study appraisal;  and *synthesis methods, such as network meta-analysis*.  Results: number of studies and participants identiﬁed; summary estimates with corresponding  conﬁdence/credible intervals; *treatment rankings may also be discussed. Authors may choose to summarize pairwise comparisons against a chosen treatment included in their analyses for brevity*.  Discussion/Conclusions: limitations; conclusions and implications of ﬁndings.  Other: primary source of funding; systematic review registration number with registry name. | Abstract |
| **INTRODUCTION** |  |  |  |
| Rationale | 3 | Describe the rationale for the review in the context of what is already known, *including mention of why a network meta-analysis has been conducted*. | Introduction |
| Objectives | 4 | Provide an explicit statement of questions being addressed, with reference to participants, interventions, comparisons, outcomes, and study design (PICOS). | Introduction |
| **METHODS** |  |  |  |
| Protocol and  registration | 5 | Indicate whether a review protocol exists and if and where it can be accessed (e.g., Web address); and, if available, provide registration information, including registration number. | Methods |
| Eligibility criteria | 6 | Specify study characteristics (e.g., PICOS, length of follow-up) and report characteristics (e.g., years considered, language, publication status) used as criteria for eligibility, giving rationale.  *Clearly describe eligible treatments included in the treatment network, and note whether any have been clustered or merged into the same node (with justiﬁcation)*. | Methods |
| Information sources | 7 | Describe all information sources (e.g., databases with dates of coverage, contact with study authors to identify additional studies) in the search and date last searched. | Methods |
| Search | 8 | Present full electronic search strategy for at least one database, including any limits used, such that it could be repeated. | Methods |
| Study selection | 9 | State the process for selecting studies (i.e., screening, eligibility, included in systematic review, and, if applicable, included in the meta-analysis). | Methods |
| Data collection process | 10 | Describe method of data extraction from reports (e.g., piloted forms, independently, in duplicate) and any processes for obtaining and conﬁrming data from investigators. | Methods |
| Data items | 11 | List and deﬁne all variables for which data were sought (e.g., PICOS, funding sources) and any assumptions and simpliﬁcations made. | Methods |
| Geometry of the network | **S1** | Describe methods used to explore the geometry of the treatment network under study and potential biases related to it. This should include how the evidence base has been graphically summarized for presentation, and what characteristics were compiled and used to describe the evidence base to readers. | Methods |
| Risk of bias within  individual studies | 12 | Describe methods used for assessing risk of bias of individual studies (including speciﬁcation of whether this was done at the study or outcome level), and how this information is to be used in any data synthesis. | Methods |
| Summary measures | 13 | State the principal summary measures (e.g., risk ratio, difference in means). *Also describe the use of additional summary measures assessed, such as treatment rankings and surface under the cumulative ranking curve (SUCRA) values, as well as modiﬁed approaches used to present summary ﬁndings from meta-analyses*. | Methods |
| Planned methods of analysis | 14 | Describe the methods of handling data and combining results of studies for each network meta-analysis. This should include, but not be limited to:  *Handling of multigroup trials;*  *Selection of variance structure;*  *Selection of prior distributions in Bayesian analyses; and Assessment of model ﬁt.* | Methods |
| Assessment of  inconsistency | **S2** | Describe the statistical methods used to evaluate the agreement of direct and indirect evidence in the treatment network(s) studied. Describe efforts taken to address its presence when found. | Methods |
| Risk of bias across studies | 15 | Specify any assessment of risk of bias that may affect the cumulative evidence (e.g., publication bias, selective reporting within studies). | Methods |
| Additional analyses | 16 | Describe methods of additional analyses if done, indicating which were prespeciﬁed. This may include, but not be limited to, the following:  Sensitivity or subgroup analyses;  Meta-regression analyses;  *Alternative formulations of the treatment network; and*  *Use of alternative prior distributions for Bayesian analyses (if applicable)*. | Methods |
| **RESULTS‡** |  |  |  |
| Study selection | 17 | Give numbers of studies screened, assessed for eligibility, and included in the review, with reasons for exclusions at each stage, ideally with a ﬂow diagram. | Results |
| Presentation of  network structure | **S3** | Provide a network graph of the included studies to enable visualization of the geometry of the treatment network. | Results |
| Summary of network geometry | **S4** | Provide a brief overview of characteristics of the treatment network. This may include commentary on the abundance of trials and randomized patients for the different interventions and pairwise comparisons in the network, gaps of evidence in the treatment network, and potential biases reﬂected by the network structure. | Results |
| Study characteristics | 18 | For each study, present characteristics for which data were extracted (e.g., study size, PICOS, follow-up period) and provide the citations. | Results |
| Risk of bias within studies | 19 | Present data on risk of bias of each study and, if available, any outcome level assessment. | Results |
| Results of individual studies | 20 | For all outcomes considered (beneﬁts or harms), present, for each study: 1) simple summary data for each intervention group, and 2) effect estimates and conﬁdence intervals. *Modiﬁed*  *approaches may be needed to deal with information from larger networks*. | Results |
| Synthesis of results | 21 | Present results of each meta-analysis done, including conﬁdence/credible intervals. *In larger*  *networks, authors may focus on comparisons versus a particular comparator (e.g., placebo or standard care), with full ﬁndings presented in an appendix. League tables and forest plots may be considered to summarize pairwise comparisons*. If additional summary measures were explored (such as treatment rankings), these should also be presented. | Results |
| Exploration for  inconsistency | **S5** | Describe results from investigations of inconsistency. This may include such information as measures of model ﬁt to compare consistency and inconsistency models, P values from statistical tests, or summary of inconsistency estimates from different parts of the treatment network. | Results |
| Risk of bias across studies | 22 | Present results of any assessment of risk of bias across studies for the evidence base being studied. | Results |
| Results of additional analyses | 23 | Give results of additional analyses, if done (e.g., sensitivity or subgroup analyses, meta-regression analyses, *alternative network geometries studied, alternative choice of prior distributions for Bayesian analyses*, and so forth). | Results |
| **DISCUSSION** |  |  |  |
| Summary of evidence | 24 | Summarize the main ﬁndings, including the strength of evidence for each main outcome; consider their relevance to key groups (e.g., health care providers, researchers, and policymakers). | Discussion |
| Limitations | 25 | Discuss limitations at study and outcome level (e.g., risk of bias), and at review level (e.g., incomplete retrieval of identiﬁed research, reporting bias). *Comment on the validity of the assumptions, such as transitivity and consistency. Comment on any concerns regarding network geometry (e.g., avoidance of certain comparisons)*. | Discussion |
| Conclusions | 26 | Provide a general interpretation of the results in the context of other evidence, and implications for future research. | Discussion |
| **FUNDING** |  |  |  |
| Funding | 27 | Describe sources of funding for the systematic review and other support (e.g., supply of data); role of funders for the systematic review. This should also include information regarding whether funding has been received from manufacturers of treatments in the network and/or whether some of the authors are content experts with professional conﬂicts of interest that could affect use of treatments in the network. | Funding |

* Boldface indicates new items to this checklist.

† Text in italics indicates wording speciﬁc to reporting of network meta-analyses that has been added to guidance from the PRISMA statement. ‡ Authors may wish to plan for use of appendices to present all relevant information in full detail for items in this section.

Article information: <https://dx.doi.org/10.21037/jtd-24-1545>

*As the checklist was provided upon initial submission, the page number/line number reported may be changed due to copyediting and may not be referable in the published version. In this case, the section/paragraph may be used as an alternative reference.

## Supplementary Digital Content 2. Details of the search strategy for the Chinese and English databases.

**Supplementary Table S2. Search strategy for the English databases.**

| **Database name** | **Search strategy** | |
| --- | --- | --- |
| PubMed  (265) | #1 | (Electric Stimulation[MeSH Terms]) OR (Electroconvulsive Therapy[MeSH Terms]) OR (Transcranial Direct Current Stimulation[MeSH Terms]) OR (Vagus Nerve Stimulation[MeSH Terms]) OR (Transcutaneous Electric Nerve Stimulation[MeSH Terms]) OR (Electrical Stimulation[Title/Abstract]) OR (Electrotherapy[Title/Abstract]) OR (neuromuscular electrical stimulation[Title/Abstract]) OR (NMES[Title/Abstract]) OR (functional electrical stimulation[Title/Abstract]) OR (FES[Title/Abstract]) OR (nerve electrical stimulation[Title/Abstract]) OR (electrical nerve stimulation[Title/Abstract]) OR (electrical muscle stimulation[Title/Abstract]) OR (electric muscle stimulation[Title/Abstract]) OR (electroconvulsive therapy[Title/Abstract]) OR (ECT[Title/Abstract]) OR (transcranial direct current stimulation[Title/Abstract]) OR (tDCS[Title/Abstract]) OR (transcranial alternating current stimulation[Title/Abstract]) OR (tACS[Title/Abstract]) OR (transcranial pulsed current stimulation[Title/Abstract]) OR (tPCS[Title/Abstract]) OR (transcranial electrical stimulation[Title/Abstract]) OR (transcranial electric stimulation[Title/Abstract]) OR (tES[Title/Abstract]) OR (transcranial electrical nerve stimulation[Title/Abstract]) OR (vagus nerve stimulation[Title/Abstract]) OR (VNS[Title/Abstract]) OR (transcutaneous vagus nerve stimulation[Title/Abstract]) OR (tVNS[Title/Abstract]) OR (transcutaneous auricular vagus nerve stimulation[Title/Abstract]) OR (taVNS[Title/Abstract]) OR (auricular vagus nerve stimulation[Title/Abstract]) OR (aVNS[Title/Abstract]) OR (galvanic vestibular stimulation[Title/Abstract]) OR (GVS[Title/Abstract]) OR (vestibular nerve stimulation[Title/Abstract]) OR (medium-frequency electrotherapy[Title/Abstract]) OR (EEG biofeedback stimulation[Title/Abstract]) OR (Stimulation electrode[Title/Abstract]) OR (Transcutaneous electrical stimulation[Title/Abstract]) OR (Transcutaneous electric stimulation[Title/Abstract]) OR (Transcutaneous electrical nerve stimulation[Title/Abstract]) OR (TENS[Title/Abstract]) OR (Percutaneous electric nerve stimulation[Title/Abstract]) OR (Transcutaneous electrical acupoint stimulation[Title/Abstract]) OR (TEAS[Title/Abstract]) OR (Transdermal electrostimulation[Title/Abstract]) OR (Electroacupuncture[Title/Abstract]) OR (Electrical acupuncture[Title/Abstract]) OR (Electric acupuncture[Title/Abstract]) OR (EA[Title/Abstract]) |
|  | #2 | (Depressive Disorders[MeSH Terms]) OR (Disorder, Depressive[Title/Abstract]) OR (Disorders, Depressive[Title/Abstract]) OR (Neurosis, Depressive[Title/Abstract]) OR (Depressive Neuroses[Title/Abstract]) OR (Depressive Neurosis[Title/Abstract]) OR (Neuroses, Depressive[Title/Abstract]) OR (Depression, Endogenous[Title/Abstract]) OR (Depressions, Endogenous[Title/Abstract]) OR (Endogenous Depression[Title/Abstract]) OR (Endogenous Depressions[Title/Abstract]) OR (Melancholia[Title/Abstract]) OR (Melancholias[Title/Abstract]) OR (Unipolar Depression[Title/Abstract]) OR (Depression, Unipolar[Title/Abstract]) OR (Depressions, Unipolar[Title/Abstract]) OR (Unipolar Depressions[Title/Abstract]) OR (Depressive Syndrome[Title/Abstract]) OR (Depressive Syndromes[Title/Abstract]) OR (Syndrome, Depressive[Title/Abstract]) OR (Syndromes, Depressive) OR (Depression, Neurotic[Title/Abstract]) OR (Depressions, Neurotic[Title/Abstract]) OR (Neurotic Depression[Title/Abstract]) OR (Neurotic Depressions[Title/Abstract]) |
|  | #3 | (((((Randomized Controlled Trial[Title/Abstract]) OR (Controlled Clinical Trial[Title/Abstract])) OR (Random[Title/Abstract])) OR (Randomization[Title/Abstract])) OR (Random Allocation[Title/Abstract])) OR (Random[Title/Abstract]) |
|  | #4 | #1 AND #2 AND #3 |

## Supplementary Digital Content 3. The list of included articles.

1.	Loo CK, Sachdev P, Martin D, Pigot M, Alonzo A, Malhi GS, et al. A double-blind, sham-controlled trial of transcranial direct current stimulation for the treatment of depression. Int J Neuropsychoph. 2010;13(1):61-9. http://doi.org/ 10.1017/ S1461145709990411

2.	Wu YH, Ning CB, Shi L, Wang JJ, Ye KH, Shen ZY, et al. A multicenter randomized clinical trial of portable transcranial alternating current stimulation for major depressive disorder. Npj Digital Medicine. 2026;9(1). http://doi.org/10.1038/ s41746-026-02575-9

3.	Boggio PS, Rigonatti SP, Ribeiro RB, Myczkowski ML, Nitsche MA, Pascual-Leone A, et al. A randomized, double-blind clinical trial on the efficacy of cortical direct current stimulation for the treatment of major depression. Int J Neuropsychoph. 2008;11(2):249-54. http://doi.org/10.1017/S1461145707007833

4.	Bulubas L, Padberg F, Bueno PV, Duran F, Busatto G, Amaro E, et al. Antidepressant effects of tDCS are associated with prefrontal gray matter volumes at baseline: Evidence from the ELECT-TDCS trial. Brain Stimul. 2019;12(5):1197-204. http://doi.org/10.1016/j.brs.2019.05.006

5.	Ranjkesh F, Barekatain M, Akuchakian S. Bifrontal versus right unilateral and bitemporal electroconvulsive therapy in major depressive disorder. J Ect. 2005;21(4):207-10. http://doi.org/10.1097/01.yct.0000187041.79087.59

6.	Kellner CH, Knapp R, Husain MM, Rasmussen K, Sampson S, Cullum M, et al. Bifrontal, bitemporal and right unilateral electrode placement in ECT: Randomised trial. Brit J Psychiat. 2010;196(3):226-34. http://doi.org/10.1192/bjp.bp.109.066183

7.	Zhou J, Li D, Ye F, Liu R, Feng Y, Feng Z, et al. Effect of add-on transcranial alternating current stimulation (tACS) in major depressive disorder: A randomized controlled trial. Brain Stimul. 2024;17(4):760-8. http://doi.org/10.1016 /j.brs. 2024 .06.004

8.	Rong P, Liu J, Wang L, Liu R, Fang J, Zhao J, et al. Effect of transcutaneous auricular vagus nerve stimulation on major depressive disorder: a nonrandomized controlled pilot study. J Affect Disorders. 2016;195:172-9. http://doi.org/ 10.1016/ j.jad.2016.02.031

9.	Du R, Zhao S, Zhang X, Li W, Ma Y, Zhang Y, et al. Efficacy and safety of lower dose electroconvulsive therapy for major depression in adolescents with suicide ideation: A non-inferiority randomized controlled trial in China. J Affect Disord. 2026;393(Pt A):120303. http://doi.org/10.1016/j.jad.2025.120303

10.	Dominiak M, Antosik-Wójcińska AZ, Goetz Z, Sikorska O, Stefanowski B, Gorostiza D, et al. Efficacy, safety and tolerability of formula-based unilateral vs bilateral electroconvulsive therapy in the treatment of major depression: a randomized open label controlled trial. J Psychiatr Res. 2021;133:52-9. http://doi.org/10.1016/ j.jpsychires.2020.12.002

11.	Liu R, Li D, Ye FK, Feng Y, Yang Z, Zhou JJ. High-intensity transcranial alternating current stimulation enhances functional connectivity of subcortical regions in major depressive disorder. J Affect Disorders. 2026;408. http://doi.org/10.1016/ j.jad.2026.121857

12.	Kang TJ, Zhao X, Huo XN, Liu X, Wang YN, Wang XL, et al. High-Intensity Transcranial Alternating Current Stimulation for Emotion Regulation and Attentional Bias in Adolescents With Nonsuicidal Self-Injury A Randomized Clinical Trial. Jama Network Open. 2026;9(6). http://doi.org/10.1001/jamanetworkopen.2026.17336

13.	Woodham RD, Selvaraj S, Lajmi N, Hobday H, Sheehan G, Ghazi-Noori AR, et al. Home-based transcranial direct current stimulation treatment for major depressive disorder: a fully remote phase 2 randomized sham-controlled trial. Nat Med. 2025;31(1):87-95. http://doi.org/10.1038/s41591-024-03305-y

14.	Borrione L, Cavendish BA, Aparicio L, Luethi MS, Goerigk S, Ramos M, et al. Home-Use Transcranial Direct Current Stimulation for the Treatment of a Major Depressive Episode: a Randomized Clinical Trial. Jama Psychiat. 2024;81(4):329-37. http://doi.org/10.1001/jamapsychiatry.2023.4948

15.	Li D, Liu R, Ye F, Li R, Li X, Liu J, et al. Modulation of brain function and antidepressant effects by transcranial alternating current stimulation in patients with major depressive disorder: evidence from ERP. J Psychiatr Res. 2024;176:1-8. http://doi.org/10.1016/j.jpsychires.2024.05.045

16.	Quan Y, Ji B, Jiang H, Fang Y, Liu YT, Guo TT, et al. Modulation of subjective and objective indicators in college students with subthreshold depression by TEAS combined with five-tone music therapy. Am J Transl Res. 2026;18(5):4492-503. http://doi.org/10.62347/ZADP1075

17.	Bennabi D, Nicolier M, Monnin J, Tio G, Pazart L, Vandel P, et al. Pilot study of feasibility of the effect of treatment with tDCS in patients suffering from treatment-resistant depression treated with escitalopram. Clin Neurophysiol. 2015;126(6):1185-9. http://doi.org/10.1016/j.clinph.2014.09.026

18.	Heikman P, Kalska H, Katila H, Sarna S, Tuunainen A, Kuoppasalmi K. Right unilateral and bifrontal electroconvulsive therapy in the treatment of depression: a preliminary study. J Ect. 2002;18(1):26-30. http://doi.org/10.1097/00124509 -200203000-00009

19.	Schwippel T, Pupillo F, LaGarde H, Stein A, Zhang MS, Rubinow DR, et al. Suppression of endogenous alpha power predicts clinical response to 10 Hz tACS in major depressive disorder: A double-blind randomized controlled trial. Brain Stimul. 2026;19(4). http://doi.org/10.1016/j.brs.2026.103131

20.	Wang H, Wang K, Xue Q, Peng M, Yin L, Gu X, et al. Transcranial alternating current stimulation for treating depression: a randomized controlled trial. Brain. 2022;145(1):83-91. http://doi.org/10.1093/brain/awab252

21.	Sharafi E, Taghva A, Arbabi M, Dadarkhah A, Ghaderi J. Transcranial Direct Current Stimulation for Treatment-Resistant Major Depression: a Double-Blind Randomized Sham-Controlled Trial. Clin Eeg Neurosci. 2019;50(6):375-82. http://doi.org/10.1177/1550059419863209

22.	Palm U, Schiller C, Fintescu Z, Obermeier M, Keeser D, Reisinger E, et al. Transcranial direct current stimulation in treatment resistant depression: a randomized double-blind, placebo-controlled study. Brain Stimul. 2012;5(3):242-51. http:// doi.org/ 10.1016/j.brs.2011.08.005

23.	Zhang T, Yu Y, Li Q, Cheng G, Hou Q, Zhang L, et al. Transcranial direct current stimulation targeting dmPFC ameliorates somatic symptoms in depression: a randomized control trial. J Affect Disorders. 2026;403:121413. http://doi.org/10.1016/ j.jad.2026.121413

24.	Jiong L, Congcong S, Weigang P, Dan W, Xiaoning S, Qian W, et al. Efficacy and Safety of Transcranial Alternating Current Stimulation Combined with Antidepressants for the Treatment of Depressive Episodes. Journal of Capital Medical University. 2022;43(2):244-8.

25.	Suli K, Cunyou G, Zilei Y, Huifeng D, Donghe L, Yunfei Z. Effects of cranial electrotherapy stimulation on the levels of HPA,BDNF and clinical symptoms in patients with mild depression Abstract. Journal of International Psychiatry. 2021;48(1):22-5.

26.	Cunyou G, Suli K, Huifeng D, Zilei Y, Donghe L. Effects of cranial electrotherapy stimulation on suicidal ideation and event-related potentials in patients with treatment-resistant depression. China Journal of Health Psychology. 2021;29(2):183-6.

27.	Chunhui H, Xueyun C, Chaoqing F, Jinyi W. Clinical research on transcranial direct current stimulation combined with votioxetine in the treatment of first-episode depression. Journal of Qiqihar Medical University. 2023;44(10):953-6.

28.	Qiong L, Shenhong W, Shihao X, Yan D. he Effects of Transcranial Direct Current Stimulation Combined with Sertraline Hydrochloride on Cognitive Function and Serum Markers in Elderly Patients with Depression. Chinese Journal of Clinical Rational Drug Use. 2022;15(22):167-70. http://doi.org/10.15887/j.cnki. 13-1389/ r.2022.22.052

29.	Xin Z, Dan W, Qian S, Wenqing J, Wen W, Yanping R. Efficacy and Safety of Different Stimulation Energy Settings in Non-Convulsive Electroconvulsive Therapy for Depressive Episodes. Journal of Jinan University（Natural Science & Medicine Edition）. 2024;45(6):589-95.

30.	Pei T, Yixiao F, Qinghua L, Du Lian, Tian Q, Haitang Q. An intervention study of right unilateral modified electroconvulsive therapy for major depressive disorder. Chinese Journal of Nervous and Mental Diseases. 2024;50(6):337-42.

31.	Wei L, Chengjun J, Kebing Y, Haipeng C, Xin W, Yongjun W, et al. Evaluation of efficacy and safety about sub-threshold modified electroconvulsive therapy for depression. Chinese Journal of Psychiatry. 2020;53(1):42-8.

32.	Zhiwen S, Zhiping X, Yaodong L, Liyan T, Lianghua D. Study of efficacy and effect on cognitive function of modified electroconvulsive therapy in treatment of depression with different electrode places. Journal of Neuroscience and Mental Health. 2010(3):274-6.

33.	Furukawa TA, Akechi T, Azuma H, Okuyama T, Higuchi T. Evidence-based guidelines for interpretation of the Hamilton Rating Scale for Depression. J Clin Psychopharm. 2007;27(5):531-4. http://doi.org/10.1097/JCP.0b013e31814f30b1

## Supplementary Digital Content 4. Electrical stimulation parameters and study designdesign

| Electrical stimulation parameters and study designdesign | | | | | |
| --- | --- | --- | --- | --- | --- |
| **Trials** | **Depression subtype/type** | **Frequency** | **Intensity** | **Pulse width** | **Sham stimulation protocol** |
| Song Zhiwen 2010 | depression | / | / | / | / |
| Luo Jiong 2022 | depression | 77. 5 Hz | 15 mA | / | A sham stimulation was employed in which the device appearance, procedure, session duration, electrode montage, and auditory/visual feedback were identical to active stimulation; except for setting the current output to 0 mA, all other intervention parameters remained unchanged to maximize blinding. |
| Kong Suli 2021 | depression | 0.5Hz | 10-50 μA | / | A sham stimulation was employed in which the device appearance, procedure, session duration, electrode montage, and auditory/visual feedback were identical to active stimulation; except for setting the current output to 0 mA, all other intervention parameters remained unchanged to maximize blinding. |
| Gao Cunyou 2021 | treatment resistant depression | 0.5Hz | 10-50 μA | / | A sham stimulation was employed in which the device appearance, procedure, session duration, electrode montage, and auditory/visual feedback were identical to active stimulation; except for setting the current output to 0 mA, all other intervention parameters remained unchanged to maximize blinding. |
| Huang Chunhui 2023 | depression | / | 2mA | / | For sham stimulation, a 2 mA current was delivered for only 40 s at each session and then immediately reduced to 0 mA; the stimulation site and all other parameters were identical to those of the intervention group. |
| Li Qiong 2022 | depression | / | 2mA | / | The device and stimulation parameters were identical to those in the observation group; however, each stimulation was limited to 40 s at a current intensity of 2 mA. |
| Zhao Xin 2024 | depression | / | / | / | / |
| Tan Pei 2024 | treatment resistant depression | / | 0.9 A | 0.5 ms | / |
| Li Wei 2020 | depression | / | / | / | / |
| Monika Dominiak 2021 | depression | / | 0.9A | 0.5ms | / |
| Elham Sharafi 2019 | treatment resistant depression | / | 2mA | / | A ramp-up–short-plateau–ramp-down pattern was used: the current was increased from baseline to 2 mA over 30 s, maintained for only 10 s, and then decreased to 0 mA over 30 s, with no current delivered for the subsequent ~20 min. This protocol mimicked skin sensations at stimulation onset and offset but provided an insufficient effective stimulation duration. |
| Peijing Rong 2016 | treatment resistant depression | 20 Hz | 4~6 mA | 0.2 ms | An anatomical non-target control was used: current intensity, stimulation duration, frequency, pulse width, and procedural steps were identical to those in the active stimulation group, but the electrodes were placed over the upper scaphoid region of the ear, an area without vagal afferent innervation, to assess the site specificity of stimulation. |
| Charles H.Kellner 2010 | treatment resistant depression | / | / | / | / |
| U. Palm 2012 | treatment resistant depression | / | 2 mA | / | All electrical parameters and procedural steps were identical to those in the active stimulation group; however, the output duration of each session was shortened to 15 s at 2 mA, resulting in markedly lower cumulative delivered charge and effective exposure duration than the active protocol. |
| Pertti Heikman 2002 | depression | / | / | / | / |
| Rui Liu 2026 | treatment resistant depression | 77.5 Hz | 15 mA | / | A sham stimulation was employed in which the device appearance, procedure, session duration, electrode montage, and auditory/visual feedback were identical to active stimulation; except for setting the current output to 0 mA, all other intervention parameters remained unchanged to maximize blinding. |
| Jingjing Zhou 2024 | treatment resistant depression | 77.5 Hz | 15 mA | / | A sham stimulation was employed in which the device appearance, procedure, session duration, electrode montage, and auditory/visual feedback were identical to active stimulation; except for setting the current output to 0 mA, all other intervention parameters remained unchanged to maximize blinding. |
| Paulo S. Boggio 2008 | treatment resistant depression | / | 2 mA | / | A ramp-up–short-plateau–ramp-down pattern was used: the current was increased from baseline to 2 mA over 30 s, maintained for only 10 s, and then decreased to 0 mA over 30 s, with no current delivered for the subsequent ~20 min. This protocol mimicked skin sensations at stimulation onset and offset but provided an insufficient effective stimulation duration. |
| Ruonan Du 2026 | treatment resistant depression | / | 800 mA | 1.0 ms | / |
| Yunhao Wu 2026 | depression | 10 Hz | 1.6mA | / | During each session, electrical stimulation was delivered for 60 s only at the initial and terminal phases, with no current output during the intermediate period; all other parameters, device interface, and operational procedures were identical to those in the active stimulation group, yielding a total effective stimulation duration of 2 × 60 s. |
| Lucia Bulubas 2019 | depression | / | 2 mA | / | The output parameters were identical to those of the active stimulation group; however, the system automatically terminated the current 30 s after onset. Following current interruption, the prompt tone, timing, electrode placement, and observation procedures were identical to active stimulation. |
| Farzad Ranjkesh 2005 | treatment resistant depression | / | / | 1.0 ms | / |
| Lucas Borrione 2024 | treatment resistant depression | / | 2 mA | / | A low-intensity 1 mA electrical signal was delivered for 45 s at the onset and offset of each stimulation to mimic the sensation of skin contact, with the output set to 0 mA during the core stimulation period. This protocol served solely as a sensory cue and did not constitute effective therapeutic stimulation. |
| Hongxing Wang 2022 | depression | 77.5 Hz | 15 mA | / | A sham stimulation was employed in which the device appearance, procedure, session duration, electrode montage, and auditory/visual feedback were identical to active stimulation; except for setting the current output to 0 mA, all other intervention parameters remained unchanged to maximize blinding. |
| Tiejun Kang 2026 | treatment resistant depression | 77.5 Hz | 15 mA | / | A sham stimulation was employed in which the device appearance, procedure, session duration, electrode montage, and auditory/visual feedback were identical to active stimulation; except for setting the current output to 0 mA, all other intervention parameters remained unchanged to maximize blinding. |
| Ye Quan 2026 | depression | 2/100 Hz | 10 mA | / | A sham stimulation was employed in which the device appearance, procedure, session duration, electrode montage, and auditory/visual feedback were identical to active stimulation; except for setting the current output to 0 mA, all other intervention parameters remained unchanged to maximize blinding. |
| Ting Zhang 2026 | depression | / | 2 mA | / | Within the 30 s immediately preceding stimulation, the current was ramped from baseline to 2 mA and then immediately returned to baseline; there was no constant-intensity plateau or sustained current output throughout the session, providing only a brief current-onset cue. |
| D. Bennabi 2015 | treatment resistant depression | / | 2 mA | / | A sham stimulation was employed in which the device appearance, procedure, session duration, electrode montage, and auditory/visual feedback were identical to active stimulation; except for setting the current output to 0 mA, all other intervention parameters remained unchanged to maximize blinding. |
| ColleenK. Loo 2010 | depression | / | 1mA | / | A sham stimulation was employed in which the device appearance, procedure, session duration, electrode montage, and auditory/visual feedback were identical to active stimulation; except for setting the current output to 0 mA, all other intervention parameters remained unchanged to maximize blinding. |
| Dan Li 2024 | treatment resistant depression | 77.5 Hz | 15 mA |  | A sham stimulation was employed in which the device appearance, procedure, session duration, electrode montage, and auditory/visual feedback were identical to active stimulation; except for setting the current output to 0 mA, all other intervention parameters remained unchanged to maximize blinding. |
| Rachel D.Woodham 2025 | treatment resistant depression | / | 2 mA | / | A sham stimulation was employed in which the device appearance, procedure, session duration, electrode montage, and auditory/visual feedback were identical to active stimulation; except for setting the current output to 0 mA, all other intervention parameters remained unchanged to maximize blinding. |
| Tobias Schwippel 2026 | treatment resistant depression | 10 Hz | 2 mA | / | A sham stimulation was employed in which the device appearance, procedure, session duration, electrode montage, and auditory/visual feedback were identical to active stimulation; except for setting the current output to 0 mA, all other intervention parameters remained unchanged to maximize blinding. |
| John Rush 2005 | treatment resistant depression | 20 Hz | 3.5 mA | 500 μs | A sham stimulation was employed in which the device appearance, procedure, session duration, electrode montage, and auditory/visual feedback were identical to active stimulation; except for setting the current output to 0 mA, all other intervention parameters remained unchanged to maximize blinding. |

## Supplementary Digital Content 5. Risk of bias assessment

| **Study ID** | **Randomization process** | **Deviations from intended interventions** | **Mising outcome data** | **Measurement of the outcome** | **Selection of the reported result** | **Overall Bias** |
| --- | --- | --- | --- | --- | --- | --- |
| Song Zhiwen 2010 | Some concerns | Some concerns | Low | Low | Low | Some concerns |
| Luo Jiong 2022 | Some concerns | Some concerns | Low | Low | Low | Some concerns |
| Kong Suli 2021 | Low | Low | Low | Low | Low | Low |
| Gao Cunyou 2021 | Some concerns | Low | Low | Low | Low | Some concerns |
| Huang Chunhui 2023 | Low | Low | Low | Low | Low | Low |
| Li Qiong 2022 | Some concerns | Low | Low | Low | Low | Some concerns |
| Zhao Xin 2024 | Low | Low | Low | Low | Low | Low |
| Tan Pei 2024 | Some concerns | Low | Low | Low | Low | Some concerns |
| Li Wei 2020 | Low | Low | Low | Low | Low | Low |
| Elham Sharafi 2019 | Low | Low | Low | Low | Low | Low |
| Peijing Rong 2016 | Low | Low | Low | Low | Low | Low |
| Charles H.Kellner 2010 | Low | Low | Low | Low | Low | Low |
| U. Palm 2012 | Low | Low | Low | Low | Low | Low |
| Pertti Heikman 2002 | Low | Low | Low | Low | Low | Low |
| Rui Liu 2026 | Low | Low | Low | Low | Low | Low |
| Jingjing Zhou 2024 | Low | Low | Low | Low | Low | Low |
| Paulo S. Boggio 2008 | Low | Low | Low | Low | Low | Low |
| Ruonan Du 2026 | Low | Low | Low | Low | Low | Low |
| Yunhao Wu 2026 | Low | Low | Low | Low | Low | Low |
| Lucia Bulubas 2019 | Low | Low | Low | Low | Low | Low |
| Farzad Ranjkesh 2005 | Low | Low | Low | Low | Low | Low |
| Lucas Borrione 2024 | Low | Low | Low | Low | Low | Low |
| Hongxing Wang 2022 | Low | Low | Low | Low | Low | Low |
| Tiejun Kang 2026 | Low | Low | Low | Low | Low | Low |
| Ye Quan 2026 | Low | Low | Low | Low | Low | Low |
| Ting Zhang 2026 | Low | Low | Low | Low | Low | Low |
| D. Bennabi 2015 | Low | Low | Low | Low | Low | Low |
| ColleenK. Loo 2010 | Low | Low | Low | Low | Low | Low |
| Dan Li 2024 | Low | Low | Low | Low | Low | Low |
| Rachel D.Woodham 2025 | Low | Low | Low | Low | Low | Low |
| Tobias Schwippel 2026 | Low | Low | Low | Low | Low | Low |
| John Rush 2005 | Low | Low | Low | Low | Low | Low |
| Monika Dominiak 2021 | Low | Low | Low | Low | Low | Low |

## Supplementary Digital Content 6. The GRADE assessment.

|  | **Direct estimate** | | **Indirect estimate** | | **NMA estimate** | |
| --- | --- | --- | --- | --- | --- | --- |
|  | **SMD(95% CI)** | **Quality of evidence** | **SMD(95% CI)** | **Quality of evidence** | **SMD(95% CI)** | **Quality of evidence** |
| **Clinical Response Rate** | | | | | | |
| Sham VS BT ECT | 1.09 (0.33,3.63) | MODERATE* | — | — | 1.09 (0.33,3.63) | MODERATE* |
| Sham VS HUL ECT | — | — | 1.10 (0.20,6.05) | LOW*⇟ | 1.10 (0.20,6.05) | LOW*⇟ |
| Sham VS BF ECT | — | — | 1.07 (0.13,8.65) | MODERATE* | 1.07 (0.13,8.65) | MODERATE* |
| Sham VS tDCS | 1.72 (1.88,3.34) | MODERATE* | — | — | 1.72 (1.88,3.34) | MODERATE* |
| Sham VS LUL ECT | — | — | 1.11 (0.20,6.25) | LOW*⇟ | 1.11 (0.20,6.25) | LOW*⇟ |
| Sham VS VNS | 5.31 (2.01,27.84) | MODERATE* | — | — | 5.31 (2.01,27.84) | MODERATE* |
| Sham VS tACS | 2.12 (0.84,5.31) | MODERATE* | — | — | 2.12 (0.84,5.31) | MODERATE* |
| tACS VS BT ECT | 0.51 (0.11,2.34) | LOW§* | — | — | 0.51 (0.11,2.34) | LOW§* |
| tACS VS HUL ECT | — | — | 0.52 (0.07,3.60) | MODERATE* | 0.52 (0.07,3.60) | MODERATE* |
| tACS VS BF ECT | — | — | 0.50 (0.05,4.96) | MODERATE* | 0.50 (0.05,4.96) | MODERATE* |
| tACS VS tDCS | — | — | 0.81 (0.26,2.52) | MODERATE* | 0.81 (0.26,2.52) | MODERATE* |
| tACS VS LUL ECT | — | — | 0.52 (0.07,3.71) | LOW*⇟ | 0.52 (0.07,3.71) | LOW*⇟ |
| tACS VS VNS | — | — | 2.51 (0.38,16.69) | LOW*⇟ | 2.51 (0.38,16.69) | LOW*⇟ |
| VNS VS BT ECT | — | — | 0.20 (0.03,1.59) | MODERATE* | 0.20 (0.03,1.59) | MODERATE* |
| VNS VS HUL ECT | — | — | 0.21 (0.02,2.23) | MODERATE* | 0.21 (0.02,2.23) | MODERATE* |
| VNS VS BF ECT | — | — | 0.20 (0.01,2.90) | MODERATE* | 0.20 (0.01,2.90) | MODERATE* |
| VNS VS tDCS | — | — | 0.32 (0.06,1.72) | MODERATE* | 0.32 (0.06,1.72) | MODERATE* |
| VNS VS LUL ECT | — | — | 0.21 (0.02,2.29) | MODERATE* | 0.21 (0.02,2.29) | MODERATE* |
| LUL ECT VS BT ECT | 0.98 (0.28,3.38) | LOW§* | — | — | 0.98 (0.28,3.38) | LOW§* |
| LUL ECT VS HUL ECT | — | — | 0.99 (0.17,5.58) | LOW*⇟ | 0.99 (0.17,5.58) | LOW*⇟ |
| LUL ECT VS BF ECT | — | — | 0.96 (0.12,7.94) | MODERATE* | 0.96 (0.12,7.94) | MODERATE* |
| LUL ECT VS tDCS | — | — | 1.55 (0.24,9.87) | LOW*⇟ | 1.55 (0.24,9.87) | LOW*⇟ |
| tDCS VS BT ECT | — | — | 0.63 (0.16,2.52) | MODERATE* | 0.63 (0.16,2.52) | MODERATE* |
| tDCS VS HUL ECT | — | — | 0.64 (0.10,4.00) | MODERATE* | 0.64 (0.10,4.00) | MODERATE* |
| tDCS VS BF ECT | — | — | 0.62 (0.07,5.60) | LOW*⇟ | 0.62 (0.07,5.60) | LOW*⇟ |
| BF ECT VS BT ECT | — | — | 1.02 (0.18,5.63) | LOW*⇟ | 1.02 (0.18,5.63) | LOW*⇟ |
| BF ECT VS HUL ECT | 1.03 (0.31,3.43) | LOW§* | — | — | 1.03 (0.31,3.43) | LOW§* |
| HUL ECT VS BT ECT | — | — | 0.99 (0.30,3.34) | MODERATE* | 0.99 (0.30,3.34) | MODERATE* |
| **HAMD Score** | | | | | | |
| Sham VS tACS | -1.35 (-2.74,0.04) | LOW§* | — | — | -1.35 (-2.74,0.04) | LOW§* |
| Sham VS HUL ECT | — | — | -1.37 (-5.01,2.27) | MODERATE* | -1.37 (-5.01,2.27) | MODERATE* |
| Sham VS BF ECT | — | — | -0.99 (-4.80,2.82) | LOW*⇟ | -0.99 (-4.80,2.82) | LOW*⇟ |
| Sham VS CES | -0.35 (-2.72,2.03) | MODERATE* | — | — | -0.35 (-2.72,2.03) | MODERATE* |
| Sham VS BT ECT | -0.86 (-4.22,2.50) | MODERATE* | — | — | -0.86 (-4.22,2.50) | MODERATE* |
| Sham VS tDCS | -1.70 (-2.80,-0.60) | LOW§* | — | — | -1.70 (-2.80,-0.60) | LOW§* |
| Sham VS LUL ECT | — | — | -0.90 (-4.71,2.91) | LOW*⇟ | -0.90 (-4.71,2.91) | LOW*⇟ |
| Sham VS VNS | 0.26 (-3.08,3.60) | LOW§* | — | — | 0.26 (-3.08,3.60) | LOW§* |
| Sham VS TEAS | -1.45 (-4.82,1.92) | MODERATE* | — | — | -1.45 (-4.82,1.92) | MODERATE* |
| TEAS VS tACS | — | — | 0.10 (-3.55,3.75) | LOW*⇟ | 0.10 (-3.55,3.75) | LOW*⇟ |
| TEAS VS HUL ECT | — | — | 0.08 (-4.88,5.04) | LOW*⇟ | 0.08 (-4.88,5.04) | LOW*⇟ |
| TEAS VS BF ECT | — | — | 0.46 (-4.63,5.55) | LOW*⇟ | 0.46 (-4.63,5.55) | LOW*⇟ |
| TEAS VS CES | — | — | 1.10 (-3.02,5.23) | LOW*⇟ | 1.10 (-3.02,5.23) | LOW*⇟ |
| TEAS VS BT ECT | — | — | 0.59 (-4.17,5.35) | MODERATE* | 0.59 (-4.17,5.35) | MODERATE* |
| TEAS VS tDCS | — | — | -0.25 (-3.80,3.29) | LOW*⇟ | -0.25 (-3.80,3.29) | LOW*⇟ |
| TEAS VS LUL ECT | — | — | 0.55 (-4.54,5.64) | LOW*⇟ | 0.55 (-4.54,5.64) | LOW*⇟ |
| TEAS VS VNS | — | — | 1.71 (-3.04,6.46) | MODERATE* | 1.71 (-3.04,6.46) | MODERATE* |
| VNS VS tACS | — | — | -1.61 (-5.23,2.01) | LOW*⇟ | -1.61 (-5.23,2.01) | LOW*⇟ |
| VNS VS HUL ECT | — | — | -1.63 (-6.57,3.31) | MODERATE* | -1.63 (-6.57,3.31) | MODERATE* |
| VNS VS BF ECT | — | — | -1.25 (-6.32,3.82) | LOW*⇟ | -1.25 (-6.32,3.82) | LOW*⇟ |
| VNS VS CES | — | — | -0.61 (-4.71,3.49) | LOW*⇟ | -0.61 (-4.71,3.49) | LOW*⇟ |
| VNS VS BT ECT | — | — | -1.12 (-5.86,3.62) | MODERATE* | -1.12 (-5.86,3.62) | MODERATE* |
| VNS VS tDCS | — | — | -1.97 (-5.48,1.55) | LOW*⇟ | -1.97 (-5.48,1.55) | LOW*⇟ |
| VNS VS LUL ECT | — | — | -1.16 (-6.23,3.91) | LOW*⇟ | -1.16 (-6.23,3.91) | LOW*⇟ |
| LUL ECT VS tACS | — | — | -0.45 (-4.50,3.60) | MODERATE* | -0.45 (-4.50,3.60) | MODERATE* |
| LUL ECT VS HUL ECT | 0.36(-1.65, 2.38) | LOW§* | 0.77(-2.60, 4.13) | LOW*⇟ | -0.47 (-2.15,1.21) | LOW§* |
| LUL ECT VS BF ECT | 0.37(-3.21, 3.95) | MODERATE* | -0.06(-2.68, 2.56) | LOW*⇟ | -0.09 (-2.15,1.97) | MODERATE* |
| LUL ECT VS CES | — | — | 0.55 (-3.93,5.04) | MODERATE* | 0.55 (-3.93,5.04) | MODERATE* |
| LUL ECT VS BT ECT | 0.16(-2.28, 2.61) | LOW§* | -0.30(-3.07, 2.48) | LOW*⇟ | 0.04 (-1.75,1.82) | LOW§* |
| LUL ECT VS tDCS | — | — | 0.36 (-1.42,2.13) | MODERATE* | -0.80 (-4.77,3.16) | MODERATE* |
| tDCS VS tACS | — | — | 0.36 (-1.42,2.13) | MODERATE* | 0.36 (-1.42,2.13) | MODERATE* |
| tDCS VS HUL ECT | — | — | 0.33 (-3.47,4.13) | LOW*⇟ | 0.33 (-3.47,4.13) | LOW*⇟ |
| tDCS VS BF ECT | — | — | 0.71 (-3.26,4.68) | MODERATE* | 0.71 (-3.26,4.68) | MODERATE* |
| tDCS VS CES | — | — | 1.36 (-1.26,3.97) | MODERATE* | 1.36 (-1.26,3.97) | MODERATE* |
| tDCS VS BT ECT | — | — | 0.84 (-2.70,4.38) | LOW*⇟ | 0.84 (-2.70,4.38) | LOW*⇟ |
| BT ECT VS tACS | — | — | -0.49 (-4.13,3.15) | LOW*⇟ | -0.49 (-4.13,3.15) | LOW*⇟ |
| BT ECT VS HUL ECT | 0.61(-0.93, 2.16) | LOW§* | -0.08(-3.74, 3.57) | LOW*⇟ | -0.51 (-1.89,0.88) | LOW§* |
| BT ECT VS BF ECT | 0.20(-2.26, 2.66) | LOW§* | 0.03(-2.78, 2.85) | MODERATE* | -0.13 (-1.93,1.67) | LOW§* |
| BT ECT VS CES | — | — | 0.52 (-3.60,4.63) | MODERATE* | 0.52 (-3.60,4.63) | MODERATE* |
| CES VS tACS | — | — | -1.00 (-3.75,1.75) | MODERATE* | -1.00 (-3.75,1.75) | MODERATE* |
| CES VS HUL ECT | — | — | -1.02 (-5.37,3.32) | LOW*⇟ | -1.02 (-5.37,3.32) | LOW*⇟ |
| CES VS BF ECT | — | — | -0.65 (-5.14,3.85) | MODERATE* | -0.65 (-5.14,3.85) | MODERATE* |
| BF ECT VS tACS | — | — | -0.36 (-4.42,3.70) | LOW*⇟ | -0.36 (-4.42,3.70) | LOW*⇟ |
| BF ECT VS HUL ECT | 0.56(-1.17, 2.29) | LOW§* | -1.03(-5.82, 3.75) | MODERATE* | -0.38 (-1.98,1.22) | LOW§* |
| HUL ECT VS tACS | — | — | -0.36 (-4.42,3.70) | MODERATE* | 0.02 (-3.87,3.91) | MODERATE* |
| **Safety** | | | | | | |
| Sham VS tACS | 1.69 (0.13,21.98) | LOW§* | — | — | 1.69 (0.13,21.98) | LOW§* |
| Sham VS BT ECT | 1.73 (0.34,8.81) | LOW§* | — | — | 1.73 (0.34,8.81) | LOW§* |
| Sham VS LUL ECT | — | — | 4.07 (0.27,60.62) | LOW*⇟ | 4.07 (0.27,60.62) | LOW*⇟ |
| Sham VS HUL ECT | — | — | 2.41 (0.44,13.01) | MODERATE* | 2.41 (0.44,13.01) | MODERATE* |
| Sham VS tDCS | 1.44 (0.82,2.52) | MODERATE* | — | — | 1.44 (0.82,2.52) | MODERATE* |
| tDCS VS tACS | — | — | 1.18 (0.09,16.24) | LOW*⇟ | 1.18 (0.09,16.24) | LOW*⇟ |
| tDCS VS BT ECT | — | — | 1.21 (0.22,6.73) | MODERATE* | 1.21 (0.22,6.73) | MODERATE* |
| tDCS VS LUL ECT | — | — | 2.83 (0.18,44.66) | LOW*⇟ | 2.83 (0.18,44.66) | LOW*⇟ |
| tDCS VS HUL ECT | — | — | 1.67 (0.28,9.91) | MODERATE* | 1.67 (0.28,9.91) | MODERATE* |
| HUL ECT VS tACS | — | — | 0.70 (0.05,10.55) | LOW*⇟ | 0.70 (0.05,10.55) | LOW*⇟ |
| HUL ECT VS BT ECT | 0.32(-0.16, 0.80) | MODERATE* | 3.86(0.23, 7.49) | MODERATE* | 0.72 (0.45,1.16) | MODERATE* |
| HUL ECT VS LUL ECT | 0.04(-3.89 , 3.89 ) | LOW§* | -0.98(-4.60 , 2.64) | MODERATE* | 1.69 (0.12,24.03) | LOW§* |
| LUL ECT VS tACS | — | — | 0.42 (0.06,3.11) | MODERATE* | 0.42 (0.06,3.11) | MODERATE* |
| LUL ECT VS BT ECT | 0.01(-3.89, 3.89) | LOW§* | 1.58(-2.01 , 5.16) | LOW*⇟ | 0.43 (0.03,5.95) | LOW§* |
| BT ECT VS tACS | — | — | 0.98 (0.07,14.31) | LOW*⇟ | 0.98 (0.07,14.31) | LOW*⇟ |
| * Serious Indirectness; ⇟ Imprecision; § Heterogeneity | | | | | | |

## Supplementary Digital Content 7. Effect-modifying characteristics

| Characteristics of included trials across interventions for assessment of transitivity. | | | | | | |
| --- | --- | --- | --- | --- | --- | --- |
| **Intervention** | **No. of RCTs (n)** | **Baseline severity (HAMD, mean±SD)** | **TRD (%)** | **Mean age (y)** | **Course of Disease(M)** | **Treatment duration** |
| HUL ECT | 8 | 33.40±6.78 | 33.33 | 40.24 | 6.64 | 5.67 |
| BF ECT | 4 | 42.25±5.78 | 66.67 | 47.03 | 11.61 | 5.00 |
| BT ECT | 6 | 27.51±7.71 | 60.00 | 38.58 | 10.02 | 5.80 |
| LUL ECT | 4 | 28.43±3.20 | 50.00 | 32.51 | 8.77 | 4.50 |
| tDCS | 13 | 24.43±4.41 | 53.85 | 43.20 | 4.47 | 4.31 |
| tACS | 6 | 21.42±4.80 | 66.67 | 32.01 | 10.75 | 3.66 |
| CES | 2 | 21.33±2.70 | 50.00 | 31.60 | 8.00 | 8.00 |
| taVNS | 1 | 24.70±5.69 | 100.00 | 40.10 | 8.90 | 8.00 |
| VNS | 1 | / | 100.00 | 47.00 | 6.60 | 10.00 |
| TEAS | 1 | / | 0.00 | 22.60 | 2.00 | 4.00 |

## Supplementary Digital Content 8. Sensitivity Analysis

**Clinical Response Rate**

**HAMD Score**

**Safety**
